# Phylogeny and host-plant relationships of the Australian Myrtaceae leafmining moth genus *Pectinivalva* (Lepidoptera, Nepticulidae), with new subgenera and species

**DOI:** 10.3897/zookeys.278.4743

**Published:** 2013-03-15

**Authors:** Robert J.B. Hoare, Erik J. van Nieukerken

**Affiliations:** 1 Landcare Research (Manaaki Whenua), Private Bag 92–170, Auckland, New Zealand (formerly Division of Botany and Zoology, Australian National University, Canberra, Australia and C.S.I.R.O. Entomology, Canberra, Australia); 2 Naturalis Biodiversity Center, PO Box 9517, NL-2300 RA Leiden, The Netherlands

**Keywords:** *Pectinivalva*, Myrtaceae, *Eucalyptus*, Paracryphiaceae, rain forest, Borneo, Indonesia, Australia, keys, hostplants, DNA barcodes

## Abstract

The phylogeny of the mainly Australian nepticulid genus *Pectinivalva* Scoble, 1983 is investigated on the basis of morphology, and a division into three monophyletic subgenera is proposed on the basis of these results. These subgenera (*Pectinivalva*, *Casanovula* Hoare, **subgen. n.** and *Menurella* Hoare, **subgen. n.** ) are described and diagnosed, the described species of *Pectinivalva* are assigned to them, and representative new species are described in each: *Pectinivalva (Pectinivalva) mystaconota* Hoare, **sp. n.**, *Pectinivalva (Casanovula) brevipalpa* Hoare, **sp. n.**, *Pectinivalva (Casanovula) minotaurus* Hoare, **sp. n.**, *Pectinivalva (Menurella) scotodes* Hoare, **sp. n.**, *Pectinivalva (Menurella) acmenae* Hoare, **sp. n.**, *Pectinivalva (Menurella) xenadelpha* Van Nieukerken & Hoare, **sp. n.**, *Pectinivalva (Menurella) quintiniae* Hoare & Van Nieukerken, **sp. n.**, and *Pectinivalva (Menurella) tribulatrix* Van Nieukerken & Hoare, **sp. n.** *Pectinivalva (Menurella) quintiniae* (from *Quintinia verdonii*, Paracryphiaceae) is the first known member of the genus with a host-plant not belonging to Myrtaceae. *Pectinivalva (Menurella) xenadelpha* from Mt Gunung Lumut, Kalimantan, Borneo, is the first pectinivalvine reported from outside Australia. Keys to the subgenera of Nepticulidae known from Australia, based on adults, male and female genitalia, and larvae, are presented. Host-plant relationships of *Pectinivalva* are discussed with relation to the phylogeny, and a list of known host-plants of *Pectinivalva*, including hosts of undescribed species, is presented. DNA barcodes are provided for most of the new and several unnamed species.

## Introduction

The subfamily Pectinivalvinae was described by Scoble (1983) for a group of Australian nepticulids retaining a pectinifer on the valva in the male genitalia. The structure of the pectinifer is similar to that in Opostegidae, the sister-group of Nepticulidae (cf. van Nieukerken 1986; Davis 1989). The subfamily remained monobasic, with *Pectinivalva* Scoble, 1983 as sole genus, until a second genus, *Roscidotoga*, was described by Hoare (2000a). *Roscidotoga* has the pectinifer absent (or possibly reduced to a thickening along the apex of the valva), but shares other apomorphies with *Pectinivalva* that support its inclusion in Pectinivalvinae (Hoare 2000a; van Nieukerken et al. 2011). The monophyly of this expanded Pectinivalvinae has been questioned by Puplesis and Robinson (1999), who provisionally follow the classification of Puplesis (1994), recognizing two subfamilies, Nepticulinae (including taxa formerly treated as Pectinivalvinae and Nepticulini) and Trifurculinae (equivalent to Trifurculini of van Nieukerken (1986)). However, Puplesis’ (1994) ‘cladogram’ is not based on cladistic principles, since he frequently lists alternative states of the same binary character as apomorphies for sister-clades (e.g., his ‘apomorphies’10–16 are merely the plesiomorphic states respectively of apomorphies 36–40, 48 and 42). Therefore this classification is rejected here, and we follow van Nieukerken (1986), Johansson et al. (1990) and Hoare (2000a) in our concepts of Pectinivalvinae and Nepticulinae. However, the current cladistic analysis is not designed to test the monophyly of Pectinivalvinae; this issue will be treated in a more comprehensive analysis of the genera and subgenera of Nepticulidae to be presented elsewhere. Ongoing molecular studies (Regier et al., van Nieukerken et al., publication pending) also confirm the monophyly of Pectinivalvinae, but not of Nepticulinae. Whereas these results may challenge the division of Nepticulidae in two subfamilies, the Pectinivalvinae will remain as a clade, and the rank of that clade is of little consequence for the present study.

Scoble (1983) recognized two subgroups within *Pectinivalva*, based on the shape of the male valva (rounded or roughly triangular). Hoare et al. (1997) made additional observations on the distinction between the two groups, which they called the *Pectinivalva commoni* and *Pectinivalva funeralis* groups, and assigned all described species to one or the other. Because of the existence of undescribed species that showed character-states of both groups, these authors did not erect a formal subgeneric distinction, in order to avoid the possibility of naming a paraphyletic taxon. The present analysis, which includes several of the ‘intermediate’ species (i.e. *Pectinivalva brevipalpa*, *Pectinivalva minotaurus*, *Pectinivalva* 219 and *Pectinivalva* 226), was designed to answer the question of whether *Pectinivalva* falls into definable monophyletic groups. In particular, is the *Pectinivalva commoni* group, which retains a number of probable plesiomorphies, monophyletic or paraphyletic with respect to the rest of the genus? To include characters of the early stages in the cladistic analysis, species were selected that had been reared, and this presented the opportunity to look at host-plant relationships in the genus from a phylogenetic perspective.

## Methods and conventions

**Preparation techniques.** Rearing techniques, and techniques of slide preparation for larvae, pupal exuviae, adult heads, wing venation and genitalia followed Hoare (2000a). In more recent preparations, phenosafranin was substituted for the acid fuchsin + azophloxin stain described by Hoare (*loc. cit*.) and no lactophenol was used.

**Morphological terminology.** Terminology follows Hoare (2000a). The term ‘androconial pocket’ is coined here for a longitudinal to elliptical furrow associated with the stem of vein Rs+M in the male hindwing of some species of *Pectinivalva*, surrounded by - usually dark - androconial scales. The pocket is presumed to function in scent production and/or dispersal during courtship, and requires further detailed investigation.

Measurements of genitalia were taken directly with a calibrated eyepiece graticule, or from photographs taken with a Zeiss Axioskop using Axiovision software (see below) and rounded off to the nearest 5μm.

Hostplant names follow the Australian Plant Census (Australian Plant Census 2012) and were checked with APNI (Australian National Botanic Gardens 2011). Hostplant classification for Myrtaceae follows Wilson (2005).

**Repositories.** The following abbreviations have been used for institutions mentioned in the text:

**anic** Australian National Insect Collection, CSIRO Entomology, Canberra, Australia.

**fmnh** Finnish Museum of Natural History, Helsinki, Finland.

**rmnh** Naturalis Biodiversity Center, Leiden, The Netherlands.

**mzb** Museum Zoologicum Bogoriense, Research Center for Biology, Indonesian Insititute of Sciences, Cibinong, Indonesia

**Material.** Type material is cited with the descriptions. All material studied, including rearing records of species that have not been treated and voucher specimens for DNA barcoding, are listed in the Excel sheet in the Online Appendix 3. We have treated specimens with a genitalia slide or other preparation and DNA vouchers as individual records for each species. Other records combine all adults from a single rearing or other single collection event. While compiling this datasheet, the authors no longer had direct access to the ANIC collection in Canberra, and in some cases our notes were not sufficient to enter all fields. This explains why for some slides ‘sex unknown’ is given, and uncertainties about exactly how many specimens were collected on a given date.

**Illustrations**. Photographs of moths by B.E. Rhode in Auckland (species described by RJBH) were taken with a Nikon Ri1 digital camera mounted on a Leica M205 A stereo-microscope. Series of images for each specimen were subsequently montaged using Helicon Focus and Zerene Stacker software packages. Post-editing work, i.e. replacing backgrounds, ‘removing’ pins, and inserting scale bars was done in Photoshop.

Photographs of moths, leafmines (jointly described species) and all genitalia slides were taken by EJvN with a Zeiss AxioCam (HR or MR5) digital camera attached respectively to a Zeiss Stemi SV11 stereo-microscope and a Zeiss Axioskop H, using Carl Zeiss AxioVision software (version 4), for some photographs using the module “Extended focus”. Manipulation of photographs, using Adobe Photoshop® was kept to a minimum: disturbing conspicuous shades, protruding parts of pins, dust and air bubbles in slides were removed or obscured, black backgrounds smoothened.

Drawings were prepared by RJBH, using a drawing tube. Drawings on one plate are not necessarily in the same scale.

**DNA barcodes.** DNA was extracted from caterpillars or from dry adult abdomens. DNA extraction from larvae was usually destructive; from abdomens and some larvae the non-destructive protocol by Knölke *et al*. (2005) was followed, allowing the preparation of the genitalia or larval skin as well. Details of methods are presented by van Nieukerken et al. (2012); we provide here the COI DNA barcode for several named and unnamed species of *Pectinivalva*, collected in 2000 and 2004 by the authors, and material on loan, collected by L. Kaila and co-workers. Barcodes of *Roscidotoga* were published before (van Nieukerken et al. 2011). Details can be found on the Barcode of Life webpages (http://www.barcodinglife.com/views/login.php ) under the project “Nepticulidae - Pectinivalvinae Public Records [NEPPP]”. Specimen data are also given in the online Appendix 3.

An NJ tree was prepared with Paup 4.0b10 for Windows (Swofford 2003), using uncorrected P distance (Srivathsan and Meier 2012). As outgroups we used *Stigmella anomalella* (Goeze, 1783) and *Opostega salaciella* (Treitschke, 1833)

**Cladistic analysis. Choice of terminal taxa:** Species were chosen with two overall aims in mind: (a) to cover the morphological diversity of *Pectinivalva* as far as possible, and (b) to cover the host-plant range as fully as possible so that inferences could be made about the evolution of host-plant choice in the genus. Only species for which both sexes of the adults were available were chosen: this criterion excluded most of the previously described species. Again, for the most part, species were chosen for which preserved larvae and mines were available; however, the early stages of *Pectinivalva caenodora* (Meyrick, 1906) are unknown. The early stages of *Pectinivalva commoni* Scoble, 1983 have not been preserved, but a very similar species (*Pectinivalva* 142: see Appendix 3) with genitalia very close to those of *Pectinivalva commoni* was reared during the course of this study and the scores for the immature stages of *Pectinivalva commoni* given here are based on the study of this second species.

Details of material examined for species not described in this paper are given in the online Appendix 3. Undescribed species which have been reared are referred to here by their generic name and a rearing number. One new species that has not beenreared was also included: this is *Pectinivalva mystaconota* sp. n. *Pectinivalva mystaconota* diverges in morphology from most typical *Pectinivalva* species in that the pectinifer on the male valva is replaced by rows of strong flattened setae (Fig. 40).

The results of an unpublished phylogenetic analysis including most subgenera of Nepticulidae (Hoare & van Nieukerken, in prep.) and molecular studies (van Nieukerken et al. in prep) suggest that *Roscidotoga* represents the sister-group of *Pectinivalva* (cf. Hoare 2000a). *Roscidotoga callicomae* Hoare, 2000 was therefore included as an outgroup, alongside a representative of Nepticulinae (*Enteucha acetosae* (Stainton, 1854)) and a representative of Opostegidae (*Notiopostega atrata* Davis, 1989).

**Cladistic analysis.** A maximum parsimony analysis with bootstrap was carried out with Paup 4.0b10 for Windows (Swofford 2003). For the heuristic search the branch swapping algorithm used was tree-bisection-reconnection (TBR). A bootstrap analysis was run for 200 replicates, each with 100 addition-sequence replicates.

Characters were traced onto the most parsimonious trees using the program Mesquite 2.7 (Maddison and Maddison 2009) to generate lists of apomorphies for monophyletic groups. Where the position of a character state change on the tree is ambiguous, the listed apomorphy is annotated according to whether it assumes accelerated transformation (ACC) or delayed transformation (DEL) of states and the alternative interpretations are given. The characters and their states are listed in Appendix 1.

## Results and list of apomorphies

**Figure 1.**
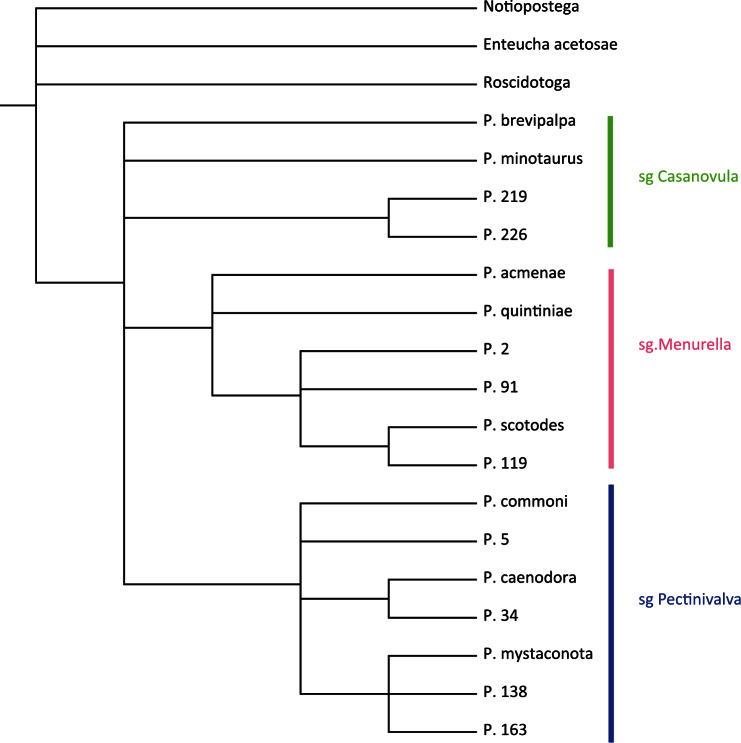
Phylogeny of Pectinivalvinae, based on 27 morphological characters of larva, pupa and adult, and 1 behavioural character of larva. Strict Consensus Tree of 147 equally most parsimonious trees (CI = 0.6667, RI = 0.8450 and RC = 0.5633). Outgroups are *Notiopostega atrata* Davis (Opostegidae), *Enteucha acetosae* (Stainton) and *Roscidotoga callicomae* Hoare (Nepticulidae).

**Figure 2.**
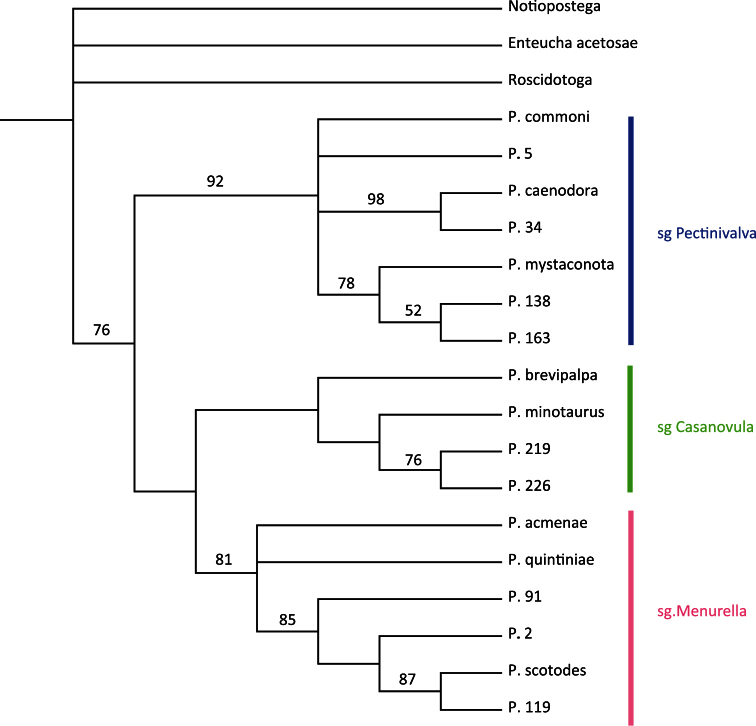
Phylogeny of Pectinivalvinae, characters and outgroups as for Fig. 1. 50% Majority Rule Consensus Tree from 147 equally most parsimonious trees; bootstrap support (200 replicates) shown on branches.

A heuristic search in PAUP produced 147 equally most parsimonious trees of length 60 with CI = 0.6667, RI = 0.8450 and RC = 0.5633. The strict consensus tree is presented in Fig. 1 (for bootstrap support see Fig. 2). *Pectinivalva* is consistently recovered as a monophyletic group with moderate support (bootstrap=76%), and within the genus, the three subgenera are almost always recovered, with strong support for *Pectinivalva* s.s. (92%) and *Menurella* (81%) (Fig. 1), but no support for *Casanovula*. *Pectinivalva brevipalpa* is the most problematic species, remaining in an unresolved polytomy with the three subgenera in the strict consensus tree. The 50% majority rule consensus tree is presented in Fig. 2, with bootstrap values; in this *Pectinivalva brevipalpa* forms a clade with *Pectinivalva minotaurus*, P. 219 and P. 226 in *Casanovula*. This placement is argued for below, and the 50% majority rule tree is used as the basis for the list of apomorphies given here, even without bootstrap support for *Casanovula*.

The monophyly of the genus *Pectinivalva* is supported by the following apomorphies:

(10–1) Uncus with a pair of well-defined tufts of setae.

(16–1) Vestibulum of female genitalia with a pair of lateral sclerites. The sclerites are absent in the clade *Pectinivalva mystaconota* + *Pectinivalva* 138 + *Pectinivalva* 163; this is unambiguously reconstructed as a secondary loss.

(17–1) Corpus bursae of female genitalia with extensive pectinations. This character is somewhat weak, since pectinations are present in many Nepticulinae (e.g. *Stigmella* and *Acalyptris* spp.). The pectinations are reduced in extent in most species of *Menurella* and lost independently in *Pectinivalva* 138 + *Pectinivalva* 163 and in *Pectinivalva quintiniae*.

(18–0 ACC) Signum of corpus bursae well-sclerotised and sparsely toothed. Since *Casanovula* and all outgroup taxa lack a signum, the basal state of this character in *Pectinivalva* is ambiguous. However, the form of the signum in *Pectinivalva* s. str. resembles that in *Pectinivalva (Menurella) acmenae* and *Pectinivalva (Menurella) quintiniae*, and similar signa do not occur elsewhere in Nepticulidae, so a single origin of this signum type is considered most likely.

(20–1) Anterior margin of T2 of abdomen with sclerotization interrupted. This character is paralleled in some genera in Trifurculini.

(23–1) Posterior lobes of larval head with sclerotization interrupted. This character is not known elsewhere in Nepticulidae. The posterior lobes are continuously sclerotized in *Pectinivalva* 119, a presumed reversion to the ancestral state.

The monophyly of *Pectinivalva* s. str. has very strong bootstrap support (92%). The group shares the following apomorphies:

(22–1) Larval head elongate and pyriform. The larva of *Pectinivalva (Menurella) brevipalpa* has a similar head-shape (Fig. 105); this is a presumed parallelism (see below).

(25–1) Larval mesothorax with only one pair of D setae (D1 absent). This character is constant within the group, but paralleled in *Pectinivalva (Casanovula)* 226 and *Pectinivalva (Menurella)* 91, and in many Nepticulinae (e.g., *Simplimorpha*, *Stigmella* spp., *Ectoedemia (Fomoria)* spp.).

(26–1) Cuticle of larva lacking spines. The larvae of all other species of *Pectinivalva* and *Roscidotoga*, and all known opostegid larvae, have a spinose cuticle. The spines are reduced or modified in some genera of Nepticulinae (e.g. the *Ectoedemia (Fomoria) vannifera* group: see Hoare 2000b), but rarely completely absent.

(27–1) Cuticle of larva with sculptured texture. This is a unique condition within the Nepticuloidea. It could be argued that characters 26 and 27 are not independent and that the sculpturing of the cuticle in *Pectinivalva* s. str. is homologous to the spines of other Nepticulidae. However, there are two reasons for rejecting this idea. Firstly, the sculpturing is particularly marked on the prothorax, where other species of Pectinivalvinae lack any spines (although some Nepticulinae have a spiny larval prothorax). Secondly, the sculpturing is present on the prothoracic sternite, an area that is never spined in other nepticulids. The two characters are therefore considered to be independent.

The following possible apomorphies are problematic:

(5–0) Forewing venation with R2+3 present. This is recovered as an unambiguous apomorphy of *Pectinivalva* s. str. in the analysis, since all other *Pectinivalva* species and all three outgroup taxa lack R2+3. However, loss of veins probably occurs rather easily in the evolution of these tiny moths, and independent losses are likely. Regaining a lost vein may not be impossible as supposed, e.g., by Meyrick (1898), but since R2+3 is present in most genera and subgenera of Nepticulinae except *Enteucha*, the character state evolution recovered here may be an artifice of taxon choice.

(15–0 DEL) Cathrema of aedeagus supported by 2 or 3 interconnected sclerites. This form of cathrema is paralleled in *Pectinivalva brevipalpa*, and therefore could equally be interpreted as an apomorphy of *Pectinivalva* as a whole, with subsequent reduction in *Pectinivalva minotaurus* + *Pectinivalva* 219 + *Pectinivalva* 226 (ACC) and fusion into a sclerotised tube in *Menurella*.

The subgenera *Menurella* and *Casanovula* together form a monophyletic group supported by the following synapomorphies (but without bootstrap support):

(9–1) Uncus bifid. The undivided, hood-like or V- or Y-shaped uncus of most Nepticulidae (including *Pectinivalva* s. str.) appears to be the plesiomorphic state in the family (cf. Scoble 1983; van Nieukerken 1986).

(21–1) Larval antenna 2-segmented (second and third segments fused). The antenna in *Pectinivalva* s. str. and in *Roscidotoga* is 3-segmented, as it is in most Lepidoptera. The basal state of this character for *Pectinivalva* is recovered as ambiguous, due to the reduced larval antennae of two of the outgroups (*Notiopostega* with two segments and *Enteucha* with one). However, independent reductions in the antenna are considered far more likely than the regaining of a lost segment, so this is regarded as a robust synapomorphy of the two groups. In *Pectinivalva quintiniae*, a further reduction to one segment has occurred, in parallel with Nepticulinae.

(24–1) Prothoracic sternite of larva much longer than broad. The Opostegidae lack a ventral prothoracic sclerotization, but most Nepticulidae, including *Roscidotoga* and *Pectinivalva* s. str., have a rather broad sclerite in this position.

If presence of vein R2+3 in *Pectinivalva* s str. is considered plesiomorphic, its loss (5–1) constitutes another probable synapomorphy of *Menurella* plus *Casanovula*, paralleled in *Roscidotoga* and *Enteucha*.

The monophyly of *Casanovula* has no bootstrap support. However, the last two of the three characters listed below are both unique within *Pectinivalva* and constant within this clade, so they are considered sufficient evidence to name this clade as a subgenus. The hostplant range is also distinctive (see below). Monophyly of the subgenus is supported by the following apomorphies:

(1–1 ACC) Basal flagellar segments of male antenna expanded and flattened. The flattened antenna occurs in *Pectinivalva brevipalpa* and *Pectinivalva minotaurus*, but not in the other two species of the group included in the analysis. Since this antennal character is unique amongst Nepticulidae, it seems very unlikely to have evolved independently in *Pectinivalva brevipalpa* and *minotaurus*, and is therefore best reconstructed as an apomorphy of *Casanovula* in the tree topology in Fig. 2, with subsequent loss in *Pectinivalva* 219 + *Pectinivalva* 226. The unresolved position of *Pectinivalva brevipalpa* in the strict consensus tree (Fig. 1) is due to the pyriform larval head (22–1), an apomorphy that it shares with *Pectinivalva* s. str. This is straightforwardly regarded as a parallelism, whereas the antennal character is not. For this reason, and because it shares the other two apomorphies listed below, *Pectinivalva brevipalpa* is confidently assigned to *Casanovula*.

(3–3) Forewing metallic, with shining fascia. Many Nepticulinae have a similar pattern, but as other species of *Pectinivalva* have drab, more or less unicolorous forewings, such coloration is apomorphic within the genus. One species of this group, not treated here (but included in the key to subgenera below) lacks a transverse fascia, but retains weakly metallic forewings with purplish reflections. Reflective coloration is known to correspond in Lepidoptera with diurnal activity, and members of this group have not been collected at light, in contrast with members of *Pectinivalva* s. str. and *Menurella*. The small eyes of the known species (interocular index less than 0.7) further confirm that the moths are diurnal.

(18–3 DEL) Corpus bursae of female genitalia lacking signum. All other species of *Pectinivalva* have a well developed signum. Although a signum is also lacking in many opostegids, including *Notiopostega*, and in the nepticulid outgroups *Roscidotoga* and *Enteucha*, the presence of a very similar form of signum (the ‘toothed band’) in *Pectinivalva* s. str. and in three plesiomorphic species of *Menurella* (*Pectinivalva acmenae*, *Pectinivalva xenadelpha* and *Pectinivalva quintiniae*) strongly suggests that the loss of the structure in *Casanovula* is apomorphic.

The monophyly of *Menurella* has strong bootstrap support (81%). Members of the group share the following apomorphies:

(12–1) Pectinifer of male valva with fewer than 20 elements. There are more than 20 elements in the pectinifer in all species of *Pectinivalva* s. str. and *Casanovula* that retain the structure, and usually more than 20 in the Opostegidae (e.g. 45 in *Notiopostega atrata*, 35 in *Eosopostega issikii* Davis, 1989). The pectinifer elements have probably been lost more than once in *Menurella* : they are absent, e.g., from *Pectinivalva quintiniae* and *Pectinivalva warburtonensis* (Wilson). Conversely, several species of *Pectinivalva* in ANIC (not treated here) clearly belong to *Menurella* on the basis of apomorphies 15–1, 16–2, 17–1 and 18–2 (see below), but have a pectinifer with more than 20 elements.

(15–1) Cathrema of aedeagus supported by a smooth sclerotized tube. The tube is always present within the group and not found in other pectinivalvines. It could possibly represent a fusion of the sclerites associated with the cathrema in *Pectinivalva* s. str. A similar smooth tubular structure is associated with the cathrema in species of *Enteucha* (see van Nieukerken 1986), although not in *Enteucha acetosae*; this is presumed to be a parallelism.

(16–2) Lateral sclerites of female vestibulum strongly developed. These sclerites are always narrow (occasionally absent) in *Pectinivalva* s. str. and *Casanovula*. In *Pectinivalva acmenae* they are still relatively narrow, but have deeply forked tips. In the remaining species of *Menurella* the sclerites are very broad and robust.

(17–1) Pectinations of corpus bursae of female restricted to posterior part of corpus. More extensive pectinations are found in most other species of *Pectinivalva* s. str. and *Casanovula* (except *Pectinivalva* 138 and *Pectinivalva* 163).

Within the three subgenera, somespecies-groups are recovered in all most parsimonious trees. Within *Pectinivalva* s. str., *Pectinivalva caenodora* and *Pectinivalva* 34 form a monophyletic unit with 98% bootstrap support (here referred to as the *Pectinivalva caenodora* group). At least one other species (*Pectinivalva* 89), not included in the current analysis, is known from this group. *Pectinivalva caenodora* and *Pectinivalva* 34 share the following apomorphies:

(3–1) Forewing with a pale costal streak. This streak is lacking in the third member of the group mentioned above.

(10–2) Tufts of setae on dorsum of uncus mounted on lobes. These lobes are found in other species of *Pectinivalva* s. str. which were not included in the current analysis and do not share the other synapomorpies of *Pectinivalva caenodora* + *Pectinivalva* 34: hence this apomorphy may define a broader species-group which includes the *Pectinivalva caenodora* group.

(11–1) Central element of gnathos broad and cordate. Shared by the third member of the *Pectinivalva caenodora* group, but again also present in a few other species of the *Pectinivalva commoni* group.

(14–1) Sublateral processes of transtilla strongly reduced.

(18–1) Signum continuously toothed.

*Pectinivalva* 163, *Pectinivalva* 138 and *Pectinivalva mystaconota* (bootstrap 78%) share the following apomorphies:

(6–1) Male hindwing strongly expanded at base. This is paralleled in *Menurella* in the group of species including *Pectinivalva scotodes*, *Pectinivalva* 2 and *Pectinivalva* 119.

(7–1) Male hindwing with androconial pocket. Again, this structure is present in the *Menurella* species listed above and in *Pectinivalva* 91.

(16–0) Vestibulum of female lacking lateral sclerites. The sclerites, which constitute an autapomorphy of *Pectinivalva*, are most parsimoniously regarded as secondarily lost in these species.

*Pectinivalva* 138 and *Pectinivalva* 163 (52% bootstrap support) share the following apomorphy:

(17–2) Pectinations of corpus bursae absent.

The position of *Pectinivalva commoni* and *Pectinivalva* 5 within *Pectinivalva* s. str. cannot be resolved on the basis of the characters used in the current analysis. *Pectinivalva commoni* most closely resembles the group formed by *Pectinivalva* 163 + *Pectinivalva* 138 + *Pectinivalva mystaconota* on the basis of head and forewing colour. It also shares with these species the presence of androconial scales on the male hindwing (Hoare et al. 1997: fig. 5), although it does not have a true androconial pocket. *Pectinivalva* 5 most closely resembles the members of the *Pectinivalva caenodora* group on the basis of the narrow valva with a strongly expanded apex (cf. Hoare et al. 1997: fig. 38): however, this could represent a plesiomorphic condition.

Within *Casanovula*, *Pectinivalva minotaurus*, *Pectinivalva* 219 and *Pectinivalva* 226 form a monophyletic group with 76% bootstrap support, based on the following synapomorphy:

(8–1) Anterior extension of vinculum H-shaped.

A further problematic synapomorphy is:

(15–2 ACC) Cathrema moderately developed, with 0–1 associated sclerites. Since this state is shared with *Roscidotoga*, the presumed sister-group of *Pectinivalva*, it may alternatively be a retained plesiomorphy (DEL) (see above, under *Pectinivalva*). In the latter case, *Pectinivalva* s. str.and *Pectinivalva brevipalpa* have independently converged on a weaker cathrema with 2–3 sclerites.

*Pectinivalva* 219 and *Pectinivalva* 226 (76% bootstrap support) share the following behavioural apomorphy:

(28–1) Exit hole a large slit; larva pupating in mine. This habit has not been observed in any other species of Pectinivalvinae.

*Pectinivalva* 226 has only one pair of D setae on the larval mesothorax (25–1); the state of this character is unknown in *Pectinivalva* 219, which was reared from pupae. It may prove to be a further apomorphy of this subgroup (paralleled in *Pectinivalva* s. str. and in P. 91).

Within *Menurella*, *Pectinivalva acmenae* and *Pectinivalva quintiniae* form an unresolved basal trichotomy with a clade containing the remaining species. The remaining species in the current analysis (*Pectinivalva scotodes*, *Pectinivalva* 2, *Pectinivalva* 91 and *Pectinivalva* 119; 85% bootstrap support) share the following apomorphies:

(7–1) Male hindwing with androconial pocket. This character is paralleled in *Pectinivalva mystaconota* + *Pectinivalva* 138 + *Pectinivalva* 163.

(13–1 ACC) Pectinifer elements of male valva broad and tooth-like. The pectinifer elements are narrow (13–0) in *Pectinivalva* 2, which is interpreted as a reversal. Alternatively, broad elements must have evolved independently in *Pectinivalva* 91 and in *Pectinivalva scotodes* + *Pectinivalva* 119.

(18–2) Signum in form of parallel spinules. This form of signum is highly characteristic of this group and not known elsewhere in Nepticulidae.

*Pectinivalva tribulatrix* (not included in the analysis) shares all these characters and also belongs to this group.

*Pectinivalva scotodes*, *Pectinivalva* 119 and *Pectinivalva* 2 form a monophyletic group supported by the following apomorphy (but without bootstrap support):

(6–1) Male hindwing strongly expanded at base. This character is paralleled in *Pectinivalva mystaconota* + *Pectinivalva* 138 + *Pectinivalva* 163 in *Pectinivalva* s. str.

*Pectinivalva scotodes* and *Pectinivalva* 119 are sister species amongst the sampled taxa (bootstrap support 87%) on the basis of the following synapomorphies:

(2–1) Sexual colour dimorphism pronounced (male forewing blackish; female forewing brown or yellowish).

(4–1) Male forewing with dorsal fringe of long narrow androconial scales.

Amongst the described species of *Pectinivalva*, *Pectinivalva funeralis* (Meyrick, 1906) and *Pectinivalva libera* (Meyrick, 1906) (each known only from the male) share apomorphy 4–1, and resemble *Pectinivalva scotodes* and *Pectinivalva* 119 in their male genitalia; they are therefore considered to belong to the same species group within *Menurella*.

### Phylogeny and classification of Pectinivalvinae: conclusions

The genus *Pectinivalva* falls into three monophyletic clades, each described and diagnosed as a subgenus of *Pectinivalva* below: subgenus *Pectinivalva* Scoble, 1983, subgenus *Casanovula* subgen. n. and subgenus *Menurella* subgen. n. It would be possible to regard these as informal species-groups. However, a more profound subdivision seems necessary, because all three subgenera contain recognizable species-groups (some of them outlined above), which are likely to be of use in the future, especially in the highly speciose subgenera *Pectinivalva* and *Menurella*. Two other possible classifications would have been consistent with the results of the cladistic analysis: (i) To give all three subgenera generic rank; (ii) to give *Pectinivalva* (*sensu stricto*) generic rank, and to erect a new genus containing *Casanovula* and *Menurella* as subgenera. Genera and subgenera are essentially artificial and subjective concepts: therefore the only criteria for judging between such classifications are considerations such as usefulness, informativeness and consistency of ranking within the family (i.e. a subgenus in *Pectinivalva* should be roughly equivalent to a subgenus in *Ectoedemia*, although sister-groups need not have the same taxonomic rank). Admittedly, the use of subgenera can be rather cumbersome; however, it also allows one to convey more information about the relationships of a species in its name. Because Scoble (1983) and van Nieukerken (1986) are followed here and elsewhere (Hoare 2000b) in recognizing subgenera in the Trifurculini, and because the degree of distinction between the three groups within *Pectinivalva* as here defined is comparable to that between trifurculine subgenera, the most conservative classification has been adopted.

The hypothesis that *Roscidotoga* falls outside *Pectinivalva*, and is not derived from within that genus, is corroborated by the cladistic analysis.

### Host-plant relationships of *Pectinivalva*

The host-plant relationships of *Roscidotoga* were discussed by Hoare (2000a) and van Nieukerken et al. (2011); their hosts are in the Cunoniaceae and Elaeocarpaceae, which belong to the Oxalidales in the eurosid I or fabid clade of the eudicots (Stevens 2008; APG III 2009). The eurosid I clade was of key significance in the early evolution of phytophagous insects (Ward et al. 2003), including Lepidoptera, and is the most important plant group in the host spectrum of Nepticulidae (Menken et al. 2010). Cunoniaceae and Elaeocarpaceae can both be considered ancient Gondwanan families based on current distribution and diversity (see discussion and references in Hoare 2000a).

As stated before, almost all known host-plants of *Pectinivalva* belong to the Myrtaceae, but one species is known from Paracryphiaceae (*Quintinia*) (see below for discussion). A full list of known host-plants is provided in Appendix 2; this includes myrtaceous hosts from which no moths have been reared, but where nepticulid mines assumed to belong to *Pectinivalva* have been collected. The assumption is based on the fact that no other genera of Nepticulidae have been reared from Myrtaceae in Australia. The Myrtaceae is an old Gondwanan family, with a long history in the southern continents (Johnson and Briggs 1981). Myrtaceae belongs to the order Myrtales in the eurosid II or malvid clade of eudicots (Stevens 2008; APG III 2009). The family reaches its greatest diversity, both at the generic and specific levels, in Australia: *Eucalyptus* L’Hérit., with approximately 800 species, dominates the vegetation over much of the continent. The dominance of Myrtaceae is certainly a relatively recent phenomenon, associated with the drying of the Australian climate during the mid to late Tertiary, and the replacement of rainforest with sclerophyllous vegetation (cf. Carpenter et al. 1994; Christophel 1994). Nevertheless, the association of *Pectinivalva* s.l. with myrtaceous hosts may predate this diversification considerably. A fossil myrtaceous leaf from the early Oligocene (ca. 35 million years B.P.) of Cethana, Tasmania (Carpenter et al. 1994: fig. 12.3e) shows an unmistakeable nepticulid mine, very similar, for example, to that of *Pectinivalva (Casanovula) brevipalpa*.

The discovery of one species of *Pectinivalva* (*quintiniae*) feeding on a host other than Myrtaceae is an interesting development. *Pectinivalva quintiniae* is in the subgenus *Menurella*, and although itapparently occupies a basal position in this subgenus (Fig. 2), it remains most parsimonious to assume that its host relationship is the result of a secondary shift from Myrtaceae. *Pectinivalva quintiniae* is a rainforest species. *Quintinia verdonii* F. Muell., the host-plant of *Pectinivalva quintiniae*, has been recently referred to the family Paracryphiaceae (APG III 2009). Winkworth et al. (2008) recovered Paracryphiaceae as sister-group to the Dipsacales, and the family now has its own order, Paracryphiales in the campanulids clade within the core eudicots (Stevens 2008; APG III 2009). Three genera are placed in the family: *Quintinia* DC., with 25 species distributed in New Guinea, Australia, New Caledonia and the Philippines, *Sphenostemon* Baill., with ten species occurring in New Guinea, Australia and New Caledonia, and *Paracryphia* Baker f., which is monotypic and endemic to New Caledonia. The distribution is consistent with a Gondwanan origin for the family, although there exists a late Cretaceous fossil from Sweden (*Silvianthemum suecicum* Friis) that has features in common with *Quintinia* (Friis 1990). Related groups, according to the phylogeny of Winkworth et al. (2008) (i.e. Dipsacales, Apiales), contain few known host-plants of Nepticulidae (van Nieukerken 1986).

Each subgenus of *Pectinivalva* has a distinctive host-plant range. Subgenus *Pectinivalva* has only been reared from species of *Eucalyptus*, the most speciose genus of Myrtaceae in Australia. *Casanovula* is the only subgenus without species on *Eucalyptus*: known host-plants are *Tristaniopsis* Peter G. Wilson, *Lophostemon* Peter G. Wilson and *Melaleuca* L. The widest range of host-plants is shown by members of *Menurella*: species have been reared from *Leptospermum* Forst. et f., *Syzygium* R.Br. ex Gaertn., *Rhodomyrtus* (DC.) Rchb., *Angophora* Cav., and *Corymbia* K.D. Hill & L.A.S. Johnson, as well as *Eucalyptus*. Leafmines found on the genera *Pilidiostigma* Burret and *Gossia* N.Snow & Guymer (both in Myrteae) and *Syncarpia* Ten. in Syncarpieae cannot yet been associated with one of the subgenera, although we predict that at least the mines on Myrteae most likely belong to *Menurella*.

In general the observation that closely-related moths tend to feed on closely-related plants (cf. van Nieukerken 1986; Hoare 2000a) is corroborated. This is clearly the case for subgenus *Pectinivalva*, where all species apparently feed on a single genus of hosts. It also applies to the *Pectinivalva (Casanovula)* 219 species group of *Casanovula*, all of whose species feed on *Melaleuca*; the inclusion of *Callistemon* R. Br. in this genus by Craven (2006) is followed here. *Pectinivalva (Casanovula) brevipalpa* and *Pectinivalva (Casanovula) minotaurus* appear very similar and closely related, although not recovered as sister-species in the cladistic analysis; however, whilst their host-plants (respectively *Tristaniopsis* and *Lophostemon*) were formerly placed together in the genus *Tristania* R. Br., recent molecular phylogenetic evidence from the matK gene suggests that they belong in different tribes of Myrtaceae (Kanieae and Lophostemoneae) (Wilson et al. 2005; Biffin et al. 2010). Finally, within subgenus *Menurella*, the group of relatively ‘derived’ species (*Pectinivalva (Menurella)* 91 + *Pectinivalva (Menurella) scotodes* Hoare + *Pectinivalva (Menurella)* 119), which have a pectinifer consisting of broad tooth-like elements, all feed on *Eucalyptus* and closely-related myrtaceous genera in the tribe Eucalypteae of Wilson et al. (2005), with the exception of *Pectinivalva (Menurella) tribulatrix*, which feeds on *Rhodomyrtus* in the tribe Myrteae. *Angophora*, the host genus of *Pectinivalva (Menurella)* 119, is sister to *Corymbia* (Hill and Johnson 1995; Wilson et al. 2005); at least one of the two (unnamed) species of *Pectinivalva* to have been reared from *Corymbia* also belongs within this ‘derived’ group of subgenus *Menurella* on the basis of the pectinifer.

The phylogeny suggests that the common ancestor of modern *Pectinivalva* was a Myrtaceae-feeder, and that the presence of a species on *Quintinia* is the result of a host-shift. It seems most likely that the split between *Roscidotoga* and *Pectinivalva* predates the Miocene aridification of Australia (ca. 24 - ca. 5 million years B.P.), and that the original hosts of *Pectinivalva* (like those of *Roscidotoga*) were rainforest plants. It is interesting that rainforest Myrtaceae still host species of subgenera *Casanovula* (*Casanovula brevipalpa* and *Casanovula minotaurus*) and *Menurella* (*Menurella acmenae*, *Menurella xenadelpha*, *Menurella quintiniae* and *Menurella tribulatrix*); the position of these species in the phylogeny is consistent with the hypothesis that their hosts may be ecologically and/or phylogenetically close to the original *Pectinivalva* host-plant.

The subgenus *Pectinivalva* appears to lack representatives on rainforest plants, and has only been reared from the sclerophyllous genus *Eucalyptus*. The earliest macrofossils currently accepted as belonging to *Eucalyptus* are leaves of *Enteucha kitsoni* Deane from the Berwick Quarry, Victoria; these date from the late Oligocene or very early Miocene (ca. 25 million years B.P.), a time when south-east Australia was probably beginning to dry climatically, though still dominated by rainforest (Pole et al. 1993). However, the clade to which *Eucalyptus* belongs (tribe Eucalypteae of Wilson et al. 2005) may have originated as long ago as the Cretaceous, as evidenced by the presence of the relictual genus *Arillastrum* Panch. ex Baillon in New Caledonia (Ladiges et al. 2003), although the relaxed molecular clock analysis by Biffin et al. (2010) gives a range of dates from late Cretaceous to Eocene for the split of Eucalypteae from Syncarpieae. A date for the split between *Pectinivalva* s.s. and the other subgenera (e.g. from molecular data) would be of great interest to shed further light on the pattern of host-plant choice.

### DNA barcodes

**Figure 3.**
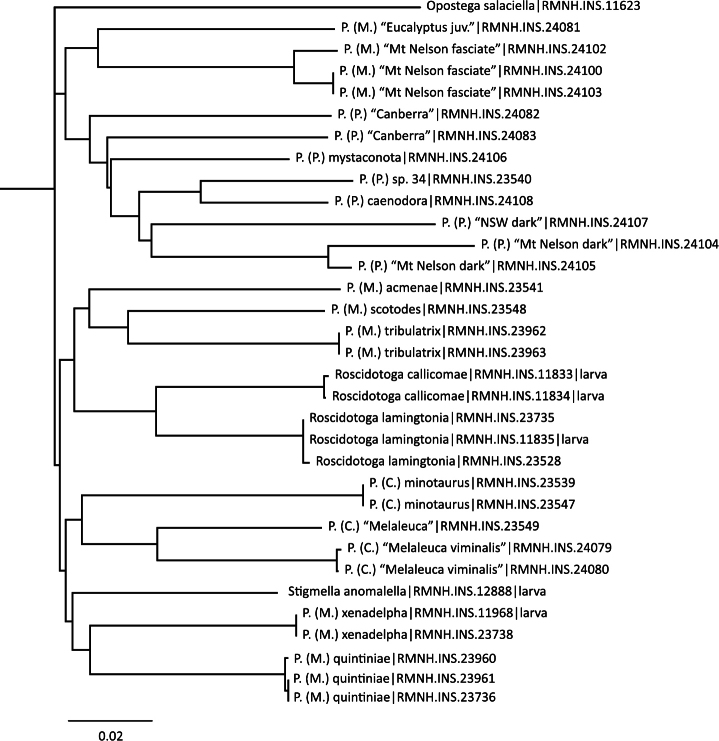
Neighbor Joining Tree of DNA barcodes of Pectinivalvinae species, showing specimen registry numbers. Outgroup is *Opostega salaciella* (Treitschke, 1833). *Stigmella anomalella* (Goeze, 1783) chosen as extra outgroup, but groups here with *Pectinivalva* subgenus *Menurella*.

We provide the DNA barcodes (Fig. 3) for seven of the eight new species named in this paper (only missing *Pectinivalva brevipalpa*), and for one previously named species (*Pectinivalva caenodora*). In addition we include barcodes for a further nine or ten unnamed species that were available for barcoding. The main reason for including barcodes is to aid recognition of species and identification of immature stages without rearing. We do not intend to use these data for phylogenetic purposes, for which data on more genes are required.

## Taxonomy

### Subfamily Pectinivalvinae

Scoble

#### Description.

Scoble (1983) gave a detailed description of the Pectinivalvinae, but recently much more material of this subfamily (especially immature stages) has become available, and the concept of the Pectinivalvinae has been expanded by Hoare (2000a) to include the previously unrecognized genus *Roscidotoga*. For these reasons a complete redescription of the subfamily is provided here. A revised diagnosis of the subfamilies of Nepticulidae is provided by Hoare (2000a: table 1).

**Table 1.** Diagnosis of the subgenera of *Pectinivalva*. Character states regarded as apomorphic are marked with an asterisk.**<br/>**

**Character**	***Pectinivalva (Pectinivalva)***	***Pectinivalva (Casanovula)***	***Pectinivalva (Menurella)***
Forewing coloration	+ unicolorous, or with costal streak	*purplish, usually with shining fascia	+ unicolorous, or with white fascia or opposite spots
Forewing venation: R2+3	Present	*Absent	*Absent
Male genitalia: uncus apex	Undivided	*Bifid	*Bifid
Male genitalia: vinculum	Rounded or weakly concave	*Strongly concave to H-shaped	Rounded to strongly concave
Male genitalia: cathrema	With 2–3 associated sclerites	Usually 1 or no associated sclerites<br/> (2–3 in *brevipalpa*)	*Supported by a sclerotized tube
Female genitalia: vestibulum	Lateral sclerites narrow or absent	Lateral sclerites narrow	*Lateral sclerites forked or broad
Female genitalia: signum	Longitudinal toothed band with lacunae	*Absent	*Oval toothed band or concentric ovals of parallel spinules
Larva: antenna	3-segmented	*2-segmented	*2-segmented or 1-segmented
Larva: D setae of mesothorax	*1 pair	2 pairs (occasionally 1)	2 pairs (occasionally 1)
Larva: spinosity of cuticle	*Spines absent	Spines present	Spines present
Larva: texture of cuticle	*Sculptured and reticulate	Smooth	Smooth

Adults. Head (Figs 19–30): Labial palpi 2- or 3-segmented; galeae short; maxillary palpi 5-segmented; antennae with sensillum vesiculocladum usually or always 5-branched (needs more detailed study in some species). Collar usually consisting of piliform scales. Forewing: underside sometimes with androconial scales in male; subdorsal retinaculum absent. Hindwing: upperside often with androconial scales in male. Wing venation (Figs 31–36): forewing without closed cell, Cu present, long; 1+2A unthickened, running obliquely from base of wing to meet dorsum before tornus; hindwing with trunk of Rs+M usually more or less deflected towards costa. Abdomen sometimes with specialized scales dorsally in male; S2a more or less pentagonal, usually with transverse rows of minute spines. Legs: fore-tibia of males sometimes thickened with specialized scales.

Male genitalia (Figs 39, 40, 42–72). Tegumen band-like, occasionally with lateral corners extended anteriorly into ‘shoulders’. Uncus either well-sclerotized and hood-like (*Pectinivalva*) or reduced (*Roscidotoga*). Gnathos (if present) with single central element. Valva usually with well-developed pectinifer. Aedeagus often with asymmetrical apical processes; striate thickening round base of ejaculatory duct (cathrema) weakly developed; vesica usually with numerous cornuti.

Female genitalia (Figs 73–103). S8 usually broadly squared off. Vestibulum often with lateral sclerites. Corpus bursae with single signum, or without signa.

Larva. Head (Figs 104–108): antennae 2- or 3-segmented (1-segmented in *Pectinivalva quintiniae*); labial palpi 3-segmented; stipes with 2 setae; frontoclypeus approximately square or rectangular; anterior tentorial arms approximately 2 times as long as posterior. Chaetotaxy (Figs 115, 116): T1 with 13 pairs of setae; T2 with 10 or 11 pairs (3 setae ventral to SV1); T3 with 9 pairs (1 D seta and 2 L setae present); A1–8 with 6 pairs of setae; A9 with 3 pairs; A10 with 3 or 4 pairs. Anal rods apically pointed or forked.

Cocoon. Usually reddish brown; usually spun outside the mine.

Pupa. Head: Clypeus squarish; frons with a pair of conspicuous setae posteriorly; labial palpi distinctly longer than maxillae. Eclosion more or less dorsal, so that suture between eyecaps and frons remains largely intact ventrally. Abdominal segments 2–8 each with 3–4 rows of spines on dorsum, and a prominent pair of dorsal setae.

#### Biology.

Most known larvae of *Pectinivalva* are leaf-miners on Myrtales (Myrtaceae), one species is known from Paracryphiales (Paracryphiaceae); those of *Roscidotoga* are leaf-miners on Oxalidales (Cunoniaceae (including Eucryphiaceae) and Elaeocarpaceae).

#### Distribution.

Australia, Borneo. Probably more widespread in Australian and Oriental regions than currently known.

##### Checklist of Pectinivalvinae

*Roscidotoga* Hoare, 2000a

*Roscidotoga eucryphiae* Hoare, 2000a

*Roscidotoga callicomae* Hoare, 2000a

*Roscidotoga lamingtonia* Van Nieukerken, Van den Berg & Hoare, 2011

*sRoscidotoga apphiripes* Hoare, 2000a

*Pectinivalva* Scoble, 1983

Subgenus *Pectinivalva (Pectinivalva)*

*Pectinivalva (Pectinivalva) caenodora* (Meyrick, 1906)

*Pectinivalva (Pectinivalva) chalcitis* (Meyrick, 1906)

*Pectinivalva (Pectinivalva) commoni* Scoble, 1983

*Pectinivalva (Pectinivalva) endocapna* (Meyrick, 1906)

*Pectinivalva (Pectinivalva) gilva* (Meyrick, 1906)

*Pectinivalva (Pectinivalva) melanotis* (Meyrick, 1906)

*Pectinivalva (Pectinivalva) mystaconota* Hoare, sp. n.

*Pectinivalva (Casanovula)* Hoare, subgen. n.

*Pectinivalva (Casanovula) brevipalpa* Hoare, sp. n.

*Pectinivalva (Casanovula) minotaurus* Hoare, sp. n.

*Pectinivalva (Menurella)* Hoare, subgen. n.

*Pectinivalva (Menurella) anazona* (Meyrick, 1906)

*Pectinivalva (Menurella) funeralis* (Meyrick, 1906)

*Pectinivalva (Menurella) libera* (Meyrick, 1906)

*Pectinivalva (Menurella) planetis* (Meyrick, 1906)

*Pectinivalva (Menurella) primigena* (Meyrick, 1906)

*Pectinivalva (Menurella) trepida* (Meyrick, 1906)

*Pectinivalva (Menurella) warburtonensis* (Wilson, 1939)

*Pectinivalva (Menurella) scotodes* Hoare, sp. n.

*Pectinivalva (Menurella) acmenae* Hoare, sp. n.

*Pectinivalva (Menurella) xenadelpha* Van Nieukerken & Hoare, sp. n.

*Pectinivalva (Menurella) quintiniae* Hoare & Van Nieukerken, sp. n.

*Pectinivalva (Menurella) tribulatrix* Van Nieukerken & Hoare, sp. n.

### Genus Pectinivalva

Scoble

http://species-id.net/wiki/Pectinivalva

Pectinivalva Scoble, 1983: 12.

#### Type species.

*Pectinivalva commoni* Scoble, 1983, by original designation.

A large and diverse genus, here subdivided into three subgenera on the basis of the phylogenetic reconstruction presented above. The following overview of the morphology of the genus should be taken in conjunction with the more detailed descriptions of the subgenera given below. Because of the great number of species in the genus, a complete revision is impractical at present. Species have been selected for description in order to represent the range of host-plants, morphology and distribution so far known in *Pectinivalva*.

#### Description.

Adults. Head capsule (Figs 19–27): labial palpi 2- or 3-segmented. Underside of forewing and upperside of hindwing often with androconial scales in male. Costal bristles of male hindwing absent or replaced by lamellate scales. Legs: fore-tibia of male sometimes thickened with specialized scales. Upperside of abdomen sometimes with androconial scales in male. Anterior edge of T2 weakly sclerotized medially.

Male genitalia (Figs 39, 40, 42–72). Anterior extension of vinculum usually rather short. Lateral arms of vinculum occasionally more or less forked apically. Uncus hood-like, dorsally with a pair of well-defined tufts of strong setae. Gnathos present, 1 central element. Valva (Figs 40, 44, 47, 50, 53, 56) rounded, squarish or triangular, usually with well-developed pectinifer along distal edge. Transverse bar of transtilla usually absent. Juxta in the form of 2 elongate sclerotized flaps connecting bases of valvae with apex of aedeagus. Aedeagus very variable (see subfamily description).

Female genitalia (Figs 73–103). S8 usually very broad and squared off. Vestibulum usually with a pair of lateral sclerites associated with apophyses anteriores. Corpus bursae well sclerotized, without diverticulum, usually with single signum.

Larva. Head (Figs 104–108): antennae 2- or 3-segmented; posterior lobes usually not continuously sclerotized caudally. Chaetotaxy (Figs 115, 116): see subgeneric descriptions.

Pupa. As described for subfamily.

#### Biology.

Most known larvae leaf-miners on Myrtaceae, with one species on Paracryphiaceae (*Quintinia* A. DC.).

#### Diagnosis.

Distinguished from *Roscidotoga*, the only other known genus of Pectinivalvinae, externally by the forewing pattern (without silver streak from mid-costa or suffusion of metallic scales towards apex); in the male genitalia by the presence of a gnathos and a well-sclerotized uncus with strong tufts of setae; and in the female genitalia by the simple (unexpanded) apophyses anteriores and the well-sclerotized corpus bursae, which lacks a diverticulum.

#### Distribution.

Australia (known from all states and territories), Borneo (a single species, *Pectinivalva xenadelpha*, described below).

### Subgenus Pectinivalva

Scoble

#### Type species:

*Pectinivalva commoni* Scoble, 1983: 13 (original designation and monotypy).

#### Description.

Adults. Head capsule (Fig. 19): labial palpi 3-segmented; interocular index 0.77–0.84. Antennae unmodified. Collar consisting of piliform scales (lamellate scales in one undescribed species). Wingspan ca. 4.5–8.4 mm. Forewing usually more or less unicolorous, greyish to fuscous, without transverse fascia, occasionally yellowish or with yellow costal streak. Hindwing in male often with longitudinal furrow (here termed ‘androconial pocket’) surrounded by androconial scales. Underside of forewing in male often with androconia. Wing venation (Figs 31, 32): R2+3 in forewing present. Abdomen usually without specialized scales; S2a always spinose. Fore-tibia of male usually thickened with blackish scales in those species with an androconial pocket.

Male genitalia (Figs 39, 40, 42, 58, 59). Lateral arms of vinculum not or weakly forked apically. Tegumen occasionally extended laterally. Valva either apically rounded, with conspicuous pectinifer of ca. 25–55 peg-like elements, or elongate and triangular, with pectinifer replaced by stiff setae. Sublateral processes sometimes reduced or absent. Aedeagus (Figs 42, 59): cathrema very weak, associated with 2 or 3 interconnected sclerites of variable length.

Female genitalia (Figs 73, 80–82). Lateral sclerites of vestibulum narrow, occasionally absent. Accessory sac absent. Signum an elongate toothed band with lacunae.

Larva. Head (Fig. 104): antennae 3-segmented, segments 2 and 3 each with 1 sensillum chaeticum and 1 sensillum basiconicum; head-shape distinctly elongate and pyriform. Thorax: prothoracic sternite (Fig. 109) broad, rounded or squarish; chaetotaxy (Fig. 115): T2 with 10 pairs of setae (1 pair of D setae). Abdomen: as described for subfamily. Cuticle of all segments completely lacking spines, and with raised reticulate texture, especially on prothorax.

#### Biology.

Host-plants: *Eucalyptus* L’Hérit. spp. (Myrtaceae). Mine: usually a short gallery leading to a blotch; exit-hole a semicircular slit.

#### Diagnosis.

See Table 1.

#### Distribution.

Australia (known from all states and territories).

#### Included species.

In addition to the six previously and newly described species, also approximately 65 undescribed species in the anic, of which the following, cited by their anic rearing numbers, have been studied in detail for the current work: *Pectinivalva (Pectinivalva)* 5; *Pectinivalva (Pectinivalva)* 34; *Pectinivalva (Pectinivalva)* 138; *Pectinivalva (Pectinivalva)* 142; *Pectinivalva (Pectinivalva)* 163.

#### Discussion.

*Pectinivalva (Pectinivalva)* is a relatively diverse subgenus, and could probably be subdivided into several species groups. One such group, the *Pectinivalva (Pectinivalva) caenodora* group, was diagnosed above. We do not propose to erect any further named species groups here, but we describe below a species that diverges strongly from most other members of the subgenus, and has several close relatives. Their placement in *Pectinivalva (Pectinivalva)* is argued for below, but as the larvae are as yet unknown, this decision may have to be revised.

*Pectinivalva (Pectinivalva)* is equivalent to the *Pectinivalva commoni* group of Hoare et al. (1997).

### Pectinivalva (Pectinivalva) mystaconota

Hoare sp. n.

urn:lsid:zoobank.org:act:D150BB4B-4D0A-41CE-830A-1B188F63DE6E

http://species-id.net/wiki/Pectinivalva_mystaconota

#### Material examined.

Holotype.♂, 35.16S, 149.06E, Black Mt., A.C.T., light trap, 26.iv.1963, I.F.B. Common. Genitalia slide 10164 (anic).Paratypes.Same locality and collector as holotype: 2♂, 28.xi.1957, 4♀, 9.xii.1957, 28.xi.1963, 14.ii.1964 and 19.ii.1964; same locality, blended light, R.J.B. Hoare: 2♂, 27.xi.1996 and 6.i.1997, slides 10161 and 12064 (anic); 6♂, Wellington, N.S.W., 28.x.1957, I.F.B. Common; 1♂, 1♀, 4 miles [6.5 km] SW of Gosford, N.S.W., 30, 31.iii.1965, I.F.B. Common, M.S. Upton; 1♂, 9 miles [14.5 km] NE of Windsor, N.S.W., 31.iii.1965, I.F.B. Common, M.S. Upton; 2♂, 220m, Mt Nelson, Hobart, Tasmania, m.v. light, 7.i.1980, 14.i.1981, P.B. McQuillan; 1♂, 42.56S 147.20E, Mt. Nelson, Tasmania, 330 m, 7.ii.2009, L. Kaila & J. Kullberg (fmnh), slides 11501–11503, 10165 (anic), EJvN 4106.

#### Description.

**Figures 4–13.**
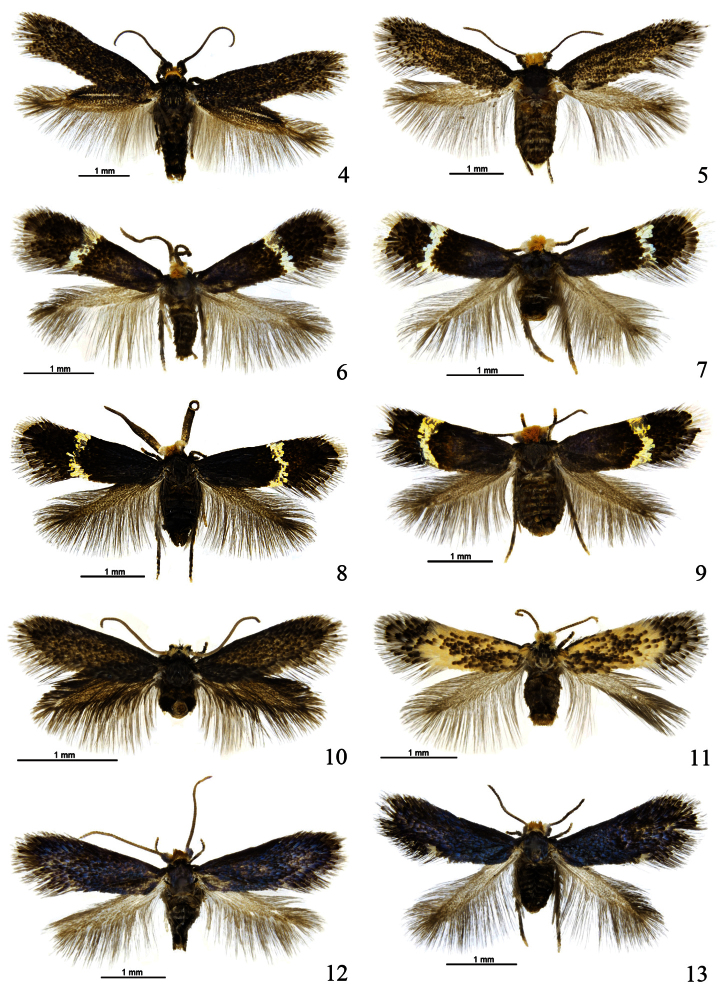
*Pectinivalva* spp., adults. **4** *Pectinivalva (Pectinivalva) mystaconota*, male paratype, Black Mt., A.C.T., 27.xi.1996 **5** *Pectinivalva (Pectinivalva) mystaconota*, female paratype, 4 miles [6.5 km] SW of Gosford, N.S.W., 30.iii.1965 **6** *Pectinivalva (Casanovula) brevipalpa*, male paratype, Fitzroy Falls, N.S.W., emg. 28.x.1996 **7** *Pectinivalva (Casanovula) brevipalpa* female paratype, Fitzroy Falls, emg. 9.xii.1996 **8** *Pectinivalva (Casanovula) minotaurus* male paratype, Leslie St., Toowoomba, Qld, emg. 8–9.ii.1996 **9** *Pectinivalva (Casanovula) minotaurus* female paratype, Leslie St., emg. 19.ii.1996 **10** *Pectinivalva (Menurella) scotodes*. male paratype, Leslie St, Toowoomba, Qld, emg. 8.x.1995 **11** *Pectinivalva (Menurella) scotodes* female paratype, McAfee’s Lookout, Brisbane Forest Park, Qld, emg. 1.x.1995 **12** *Pectinivalva (Menurella) acmenae* male paratype, Mt Dromedary, N.S.W., emg. 22.x.1995 **13** *Pectinivalva (Menurella) acmenae* female paratype, Kioloa State Forest, N.S.W., emg. 11.x.1995.

Male (Fig. 4). Wingspan 5.8–7.6 mm. Head capsule: labial palpi distinctly longer than galeae; maxillary palpi with ratio of segments from base approximately 0.3: 0.4: 0.6: 1.5: 1.0; interocular index 0.74. Frontal tuft orange; collar inconspicuous, white; eyecaps black, thinly scaled and almost transparent towards base; antennae blackish, 37–42 segments. Thorax and forewing blackish fuscous, weakly shining; cilia concolorous. Hindwing broadened at base, clothed in dark brown scales with iridescent reflections; an elongate androconial pocket in anterior ½ of wing, surrounded by shining granular blackish scales; cilia dark grey. Underside: forewing and hindwing dark fuscous; costa of hindwing with a series of blunt rectangular lamellate scales. Wing venation as in Fig. 31: base of R1 in forewing well separated from base of R2+3; trunk of Rs+M in hindwing not strongly deflected towards costa. Legs: fore-tibia somewhat thickened with blackish scales. Abdomen dark fuscous, with a moustache-like patch of hair-scales on T5 (Fig. 41).

Female (Fig. 5). Wingspan 7.5–8.0 mm. Similar to male, but head broader; antennae shorter, 30 segments; forewing somewhat broader; hindwing unmodified. Wing venation as in Fig. 32: base of R1 in forewing close to base of R2+3; trunk of Rs+M in hindwing strongly deflected towards costa. T5 of abdomen without hair-scales.

Male genitalia (Figs 39, 40, 42, 58, 59). Capsule ca. 480 μm long. Vinculum with anterior margin W-shaped; lateral arms and tegumen forming a triangle. Tegumen very narrow, caudally rounded. Uncus hood-like with well-sclerotized tip. Gnathos with enlarged basal plate, lateral arms slightly curved, central element short, triangular. Valva (Fig. 40) ca. 280–335 μm long, triangular and pointed; a spine-like process at base of medial edge; inner (dorsal) surface with numerous strong flattened setae in apical ½; exterior surface with a tuft of very robust, long setae extending beyond tip of valva (visible without dissection); sublateral processes well developed; pectinifer absent. Aedeagus (Figs 42, 59) ca. 550–575 μm including processes; 3 large blunt interconnected processes at apex, the left hand one curved; vesica with a large field of small cornuti, cathrema with 3 loosely interconnected elongate sclerites.

Female genitalia (Figs 73, 80–82). Total length ca. 935 μm. T9 not forming distinct anal papillae, 8–9 setae on each side. T8 with ca. 9–10 setae on each side. Apophyses anteriores slightly longer than posteriores (Fig. 81). Lateral sclerites of vestibulum absent. Corpus bursae with elongate posterior portion and oval anterior portion; anterior portion with strong transverse folds and numerous strong close-set pectinations. Signum (Fig. 82) an elongate weakly toothed band along anterior edge of corpus.

**Figures 14–18.**
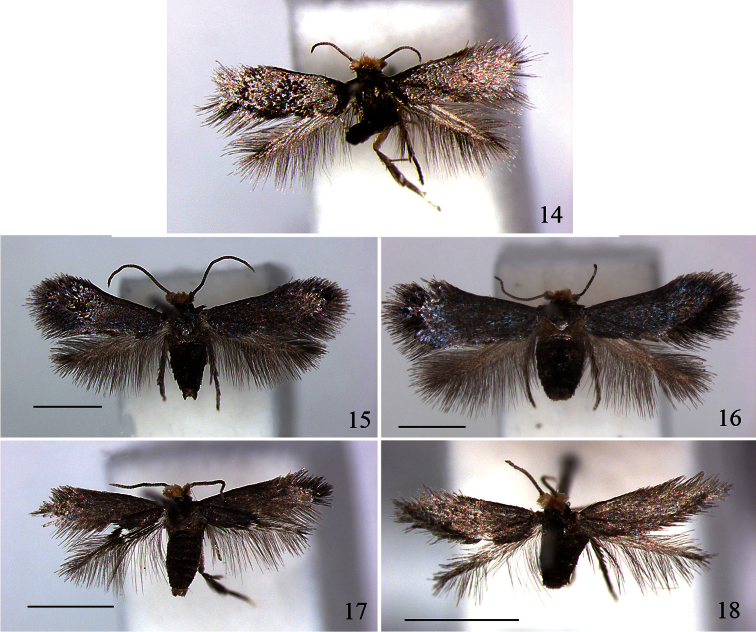
*Pectinivalva (Menurella)* spp., adults. **14** *Pectinivalva (Menurella) xenadelpha*, female holotype, Indonesia, Kalimantan, Gunung Lumut, emg. 15.xii.2005 **15** *Pectinivalva (Menurella) quintiniae*, male holotype, Tullawalal, Lamington N.P., Qld, emg. 25.ix.-6.x.2004 **16** *Pectinivalva (Menurella) quintiniae*, female paratype, Tullalwalal, emg. 25.ix.–6.x.2004 **17** *Pectinivalva (Menurella) tribulatrix*, male holotype, Cape Tribulation, Qld, emg. 8.ix.2004 **18** *Pectinivalva (Menurella) tribulatrix*, female paratype, Cape Tribulation, emg. 8.ix.2004. Scales 1 mm.

#### Diagnosis.

Both sexes can be distinguished from similar members of the subgenus by the black eyecaps with their weakly scaled transparent bases. In addition, the combination of the pointed valvae with their long, strong setae (visible in undissected males) and the moustache-like patch of hair-scales on T5 of the abdomen is characteristic of the male. In the female genitalia, the absence of lateral sclerites in the vestibulum and the transversely rugose corpus bursaeare diagnostic.

#### Distribution.

Collected in scattered localities in eastern Australia from Wellington, N.S.W. south to Mt Nelson, Hobart, Tasmania; presumably widespread, but not yet known from Victoria.

#### DNA barcode.

RMNH.INS.24106, Genbank KC292479

#### Derivation.

The specific name is derived from the Greek *mystax* (a moustache) and *notos* (a back) and refers to the tuft of hair-scales on T5 in the male. It is an adjective.

#### Remarks.

Several species related to *Pectinivalva (Pectinivalva) mystaconota* are known: all lack a pectinifer and have more or less dense tufts of setae on the dorsal surface of the valva. The group seems to be best represented in Western Australia. As the larvae are unknown, and the only definite apomorphies for the subgenus *Pectinivalva* are characters of the larva, the assignment of this group to the subgenus remains to be confirmed. The undivided uncus and the form of the sclerites associated with the cathrema in the male genitalia, and the form of the signum in the female genitalia, are characteristic of the subgenus *Pectinivalva*, but these features may be plesiomorphic within the genus as a whole. However, in the most parsimonious trees resulting from the cladistic analysis presented above, *Pectinivalva mystaconota* was placed as sister species to *Pectinivalva (Pectinivalva)* 138 + *Pectinivalva (Pectinivalva)* 163. For these reasons, it is here placed in the subgenus *Pectinivalva*.

### Subgenus Casanovula

Hoare subgen. n.

#### Type species.

*Pectinivalva (Casanovula) brevipalpa* sp. n.

#### Description.

Adults. Head capsule (Figs 23–30): labial palpi either normal, 3-segmented, or with segments 2 and 3 reduced (Fig. 27), or 2-segmented (Fig. 26); interocular index 0.55–0.68. Antennae sometimes dilated and flattened at base. Head colour blackish or orange; eyecaps white, often blackish posteriorly. Collar consisting of piliform scales. Wingspan 3.8–6.0 mm. Forewing with dark blue or purplish lustre and (except in one species) transverse shining silver or pale gold fascia. Hindwing without androconial pocket. Underside of male forewing without androconia. Wing venation (Fig. 33): R2+3 in forewing absent. Upperside of abdomen in male sometimes with specialized lamellate scales; S2a strongly spinose or without spines. Legs: fore-tibia of male not thickened above with scales.

**Figures 19–27.**
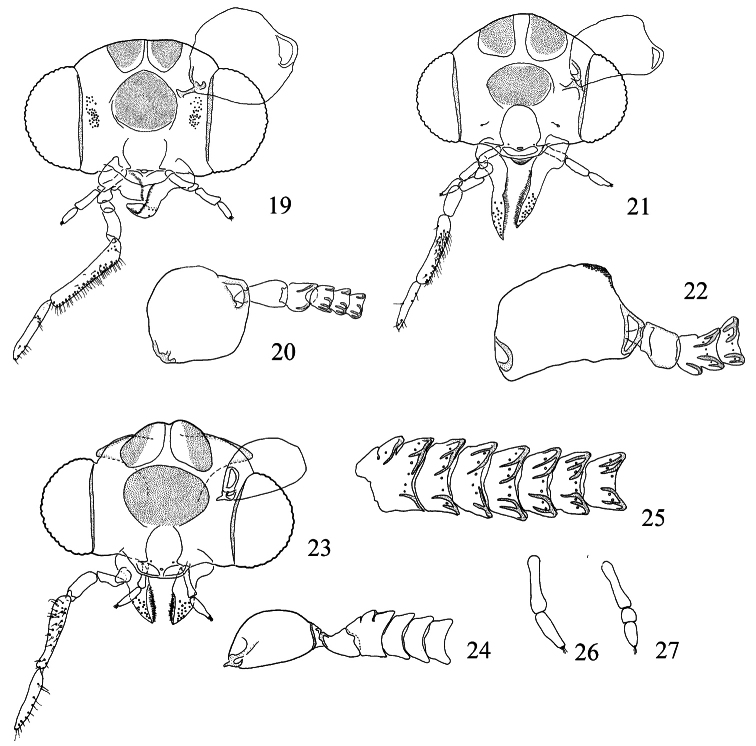
*Pectinivalva* spp, adult male heads, anterior view. **19** *Pectinivalva (Pectinivalva)* 138, head **20** *Pectinivalva (Pectinivalva)* 138 antennal base **21** *Pectinivalva (Menurella) scotodes*, head **22** *Pectinivalva (Menurella) scotodes* antennal base **23–26** *Pectinivalva (Casanovula) brevipalpa*: **23** head **24** antennal base **25** flagellomeres 1–7, showing sensillum vesiculocladum **26** labial palpus **27** *Pectinivalva (Casanovula) minotaurus* labial palpus.

**Figures 28–30.**
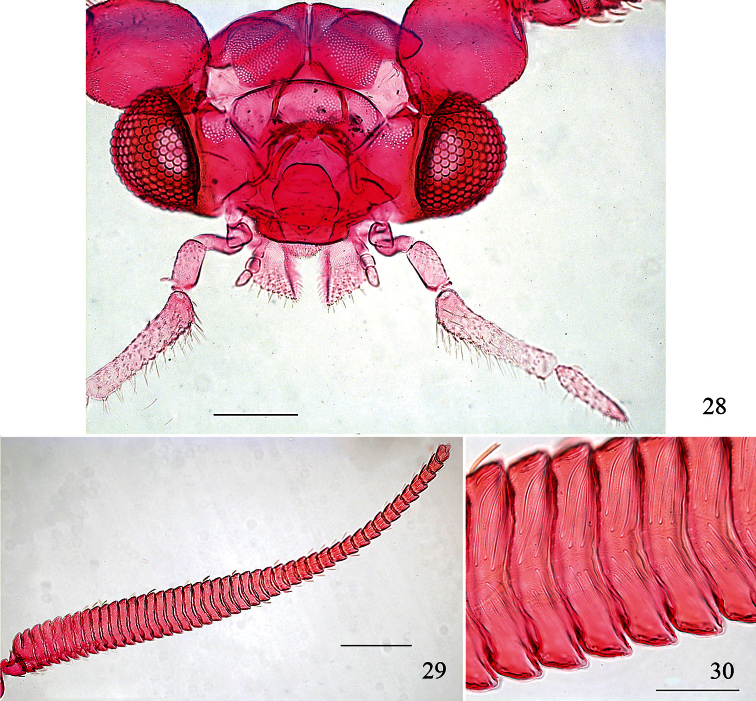
*Pectinivalva (Casanovula) minotaurus*, adult male head, anterior view. **28** Head **29** whole antenna, excluding scape **30** portion of basal ½ of flagellum, showing sensillum vesiculocladum. All from slide ANIC11325. Scales 100 μm (28), 200 μm (29), 50 μm (30).

**Figures 31–36.**
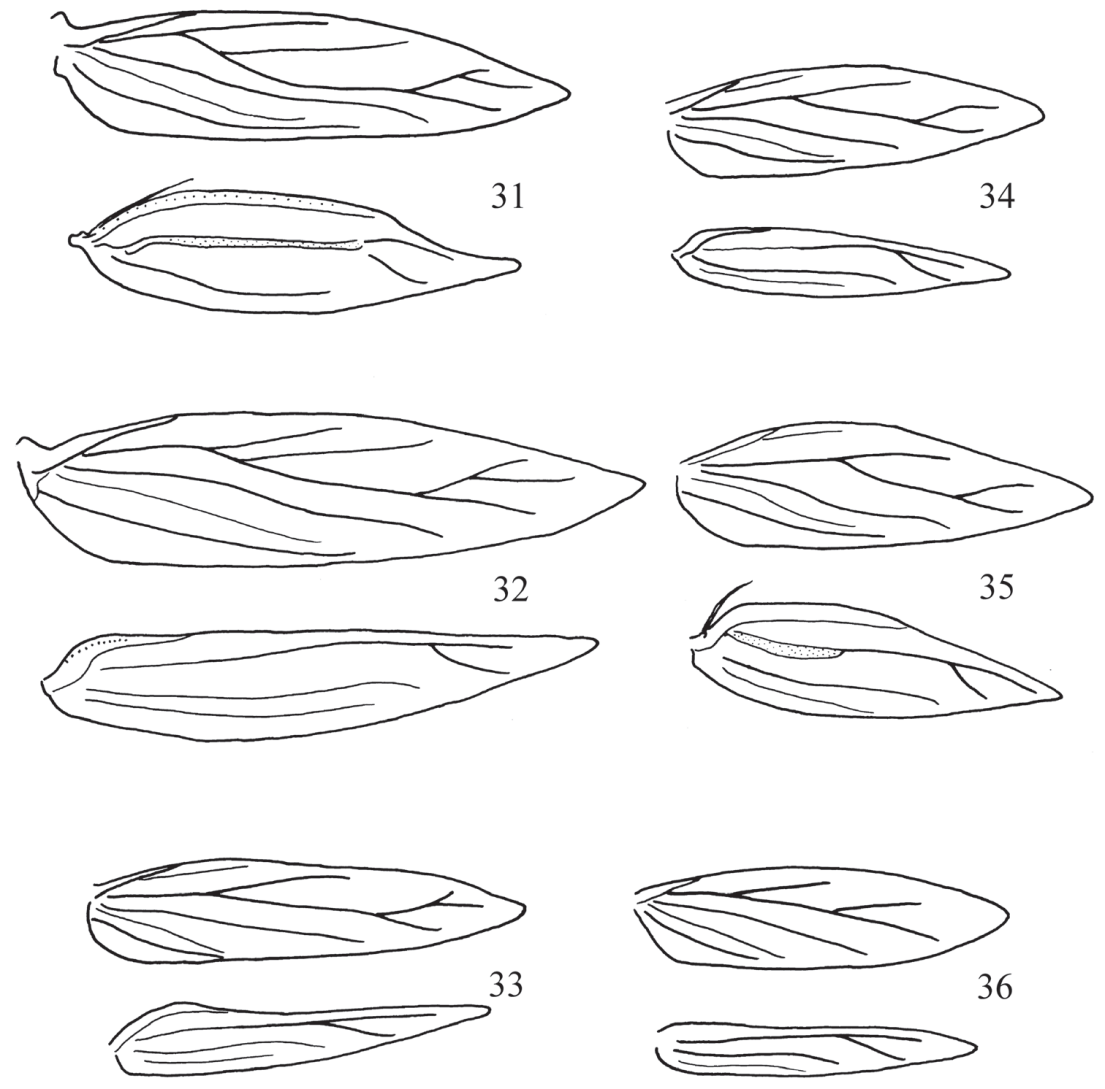
*Pectinivalva* spp., wing venation. **31** *Pectinivalva (Pectinivalva) mystaconota*, male **32** *Pectinivalva (Pectinivalva) mystaconota*, female **33** *Pectinivalva (Casanovula) brevipalpa* male **34** *Pectinivalva (Menurella) scotodes* female **35** *Pectinivalva (Menurella) scotodes* male **36** *Pectinivalva (Menurella) acmenae* female.

**Figures 37, 38.**
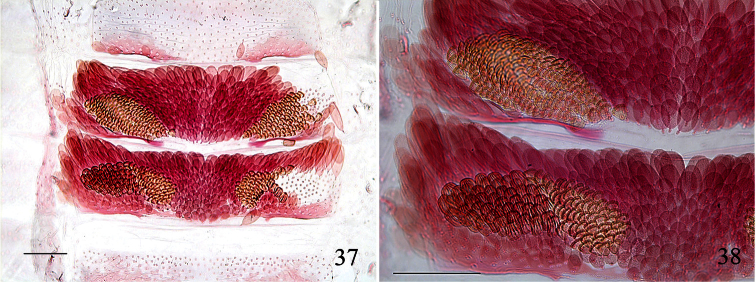
*Pectinivalva (Casanovula) minotaurus*, male androconia. **37** Abdominal tergites 4–5, partially descaled **38** close-up of androconia on one side of T4–5, showing the two distinct types. Slide ANIC11325, scales 100 μm.

Male genitalia(Figs 43–48, 60–63). Anterior extension of vinculum more or less H-shaped, with strong medial excavation. Tegumen simple. Uncus apically bifid, basally with 2 weakly sclerotized areas. Gnathos central element narrow and pointed. Valva (Figs 44, 47) relatively stout, apically rounded or squared off, pectinifer consisting of ca. 22–29more or less peg-like elements. Aedeagus (Figs 45, 48, 61, 63): vesica with numerous small cornuti; cathrema moderately weak, with or without associated sclerites.

Female genitalia (Figs 74, 75, 83–88). Lateral sclerites of vestibulum present, narrow. Accessory sac more or less developed. Corpus bursae with numerous pectinations, signum absent.

Larva. Head (Figs 105, 106): antennae 2-segmented; segment 2 with 1 pair of sensilla chaetica and 1 pair of sensilla basiconica; head-shape cordate or pyriform. Thorax: prothoracic sternite (Figs 110, 111) more or less narrow, subtriangular; chaetotaxy (Fig. 116): T2 with 10 or 11 pairs of setae (2 pairs of D setae (except in *Pectinivalva (Casanovula)* 226, which has 1 pair of D setae); L3 present or absent). Abdomen: as described for subfamily. Cuticle not textured, all segments except T1 and A10 with covering of fine spines.

**Figures 39–48.**
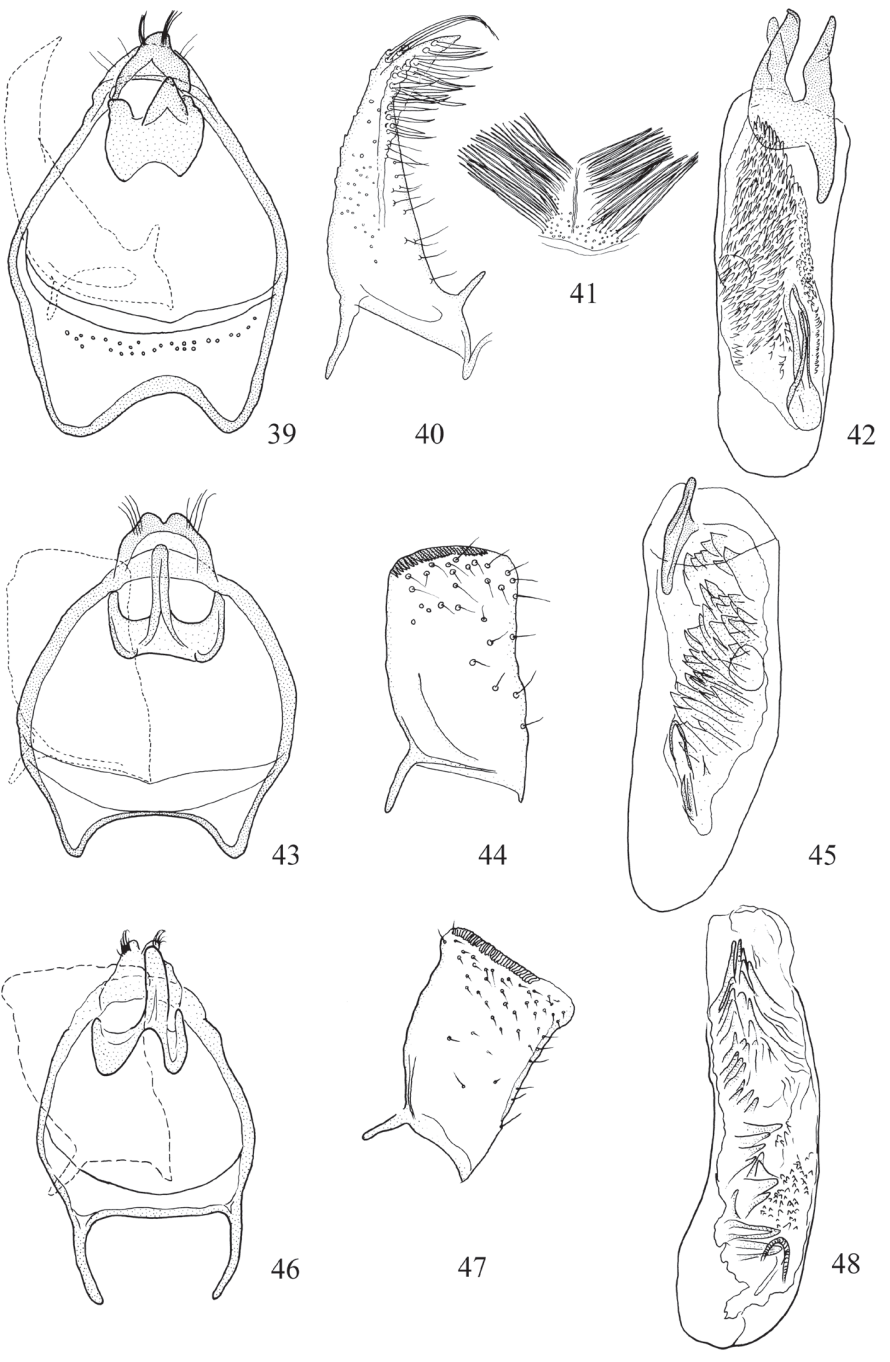
*Pectinivalva* spp., male androconia and genitalia, ventral view. **39–42** *Pectinivalva (Pectinivalva) mystaconota*: **39** genital capsule **40** left valva **41** androconial scales on T5 **42** aedeagus **43–45** *Pectinivalva (Casanovula) brevipalpa*: **43** genital capsule **44** left valva **45** aedeagus **46–48** *Pectinivalva (Casanovula) minotaurus*: **46** genital capsule **47** left valva **48** aedeagus.

#### Biology.

Host-plants: *Lophostemon* Peter G. Wilson spp., *Tristaniopsis* Peter G. Wilson spp., and *Melaleuca* L. spp. (including species formerly assigned to *Callistemon* R. Br.) (all Myrtaceae). Mine (Figs 117, 118): either a narrow gallery more or less filled with frass, or a gallery expanding into a blotch; exit-hole a small semicircular slit, a small semicircular hole, or a large slit: in the last case larva pupating in mine.

#### Diagnosis.

See Table 1.

#### Distribution.

Known only from eastern Australia: Queensland, N.S.W., A.C.T. and Tasmania; to be expected in Victoria.

#### Derivation.

The subgenus is named (in the diminutive) after the famous Italian adventurer and philanderer Giacomo Casanova, in reference to the unusual sexual ornamentation of the males of some species (e.g. *Pectinivalva (Casanovula) minotaurus* sp. n., in which the male has strongly dilated antennae and specialized scales on the abdomen upperside). It is considered feminine (in spite of its derivation) to accord with the gender of *Pectinivalva*.

#### Included species.

No previously described species are referable to this subgenus. The following species are described below: *Pectinivalva (Casanovula) brevipalpa* sp. n. and *Pectinivalva (Casanovula) minotaurus* sp. n. Also at least five undescribed species in the anic, of which the following, cited by their anic rearing numbers, have been studied in detail for the current work: *Pectinivalva (Casanovula)* 219; *Pectinivalva (Casanovula)* 226.

#### Discussion.

This is the least speciose of the three subgenera of *Pectinivalva*. Two species-groups can conveniently be recognised. In the *Pectinivalva (Casanovula) brevipalpa* group (not monophyletic according to the cladistic analysis), the antenna of the male is dilated and flattened at the base,the vertex bears a pair of sclerotized crests, and the labial palpus is modified, with segments 2 and 3 reduced or fused. Abdominal sternite 2a is strongly spinose. The host-plants are *Tristaniopsis* and *Lophostemon* spp. and pupation is outside the mine. In addition to *Pectinivalva brevipalpa* and *Pectinivalva minotaurus*, a single undescribed species (*Pectinivalva* 41, feeding on *Lophostemon confertus*), is referable to this species group. In the *Pectinivalva (Casanovula)* 219 group, the labial palpi and the vertex are unmodified; S2a lacks spines; the host-plants belong to *Melaleuca* (including *Callistemon*), and pupation may be within the mine. About four species are known in this species group.

### Pectinivalva (Casanovula) brevipalpa

Hoare sp. n.

urn:lsid:zoobank.org:act:E17B15B1-A20D-4AE6-A4B6-C4F3E970640B

http://species-id.net/wiki/Pectinivalva_brevipalpa

#### Material examined.

Holotype. ♂, 34.39S, 150.29E, Fitzroy Falls, N.S.W., emg. 25.x.1996, *Tristaniopsis collina*, R.J.B. Hoare. Genitalia slide 12111 (anic). Paratypes. 6♂, 5♀, same data as holotype, emg. 17.x.-9.xii.1996; 2♂, 4♀, 34.48S, 150.34E, Cambewarra Lookout, N.S.W., *Tristaniopsis collina*, emg. 15.x.-22.xii.1995, R.J.B. Hoare and E.S. Nielsen; 1♂, 1♀, 35.37S, 150.16E, 1 km SE of East Lynne, Kioloa State Forest, N.S.W., emg. 26, 28.x.1995, *Tristaniopsis collina*, R.J.B. Hoare; 1♀, Clyde Mt., N.S.W., [host unidentified], emg. 28.x.1963, I.F.B. Common. Slides 11237, 11238, 11328, 12065, 12136 (anic).

#### Description.

Male (Fig. 6). Wingspan 4.3–5.9 mm. Head capsule (Figs 23–26): labial palpi reduced, 2-segmented; maxillary palpi with ratio of segments from base approximately 0.2: 0.4: 0.5: 1.2: 1.0; interocular index 0.67; vertex with a pair of sclerotized crests. Frontal tuft ferruginous; collar white; eyecaps white; antennae with basal segments dilated and flattened, gradually tapering, shining lead-grey, whitish beneath, ca. 32 segments. Thorax and tegulae dark fuscous with purplish reflections. Forewing to 2/3 dark fuscous with purplish reflections; a shining silver to pale golden fascia at 2/3, slightly broader on costa, apex of wing dark fuscous without reflections; cilia pale grey beyond a line of fuscous-tipped scales. Hindwing grey, unmodified; cilia grey. Abdomen lead-grey, slightly shining.

Female (Fig. 7). Wingspan 4.3–5.2 mm. Similar to male, but antennae not dilated at base, 18 segments.

Male genitalia (Figs 43–45, 60, 61). Capsule ca. 250 μm. Anterior extension of vinculum with semicircular excavation. Uncus squarish, bilobed, with a tuft of 5–6 setae arising from dorsal side of each lobe near tip, centre of uncus with two weakly sclerotized ‘windows’. Gnathos with elongate central element and short lateral arms. Valva (Fig. 44) ca. 190 μm, squarish; pectinifer consisting of ca. 27 narrow elements. Transtilla absent. Aedeagus (Fig. 45, 61) 360 μm, a single rather broad, blunt spine at apex on left. Vesica with numerous close-set rather broad cornuti.

Female genitalia (Fig. 74, 83–85). Total length 520 μm. T9 with 7 setae on each side. Apophyses anteriores rather narrow with slightly incurved tips; apophyses posteriores slightly narrower and longer than anteriores. Lateral sclerotizations of vestibulum narrow, bent inwards, tips unmodified. Ductus spermathecae with 1 indistinct convolution. Posterior part of corpus bursae very convoluted; anterior part with many coarse pectinations; 2 or 3 indistinct elongate sclerotizations ½ way down corpus.

Larva. Green. Head (Fig. 105) elongate, pyriform; length of head ca. 410 μm; width ca. 295 μm. Thorax: prothoracic sternite as in Fig. 110. Chaetotaxy (Fig. 116) as described for subgenus; T2 with 10 pairs of setae (L3 absent); A10 with 4 pairs. Anal rods distinctly forked posteriorly.

#### Biology.

Host plant: *Tristaniopsis collina* Peter G. Wilson & Waterhouse (Myrtaceae). Egg: on underside of leaf. Mine (Fig. 117): commences as very long narrow gallery either filled with greenish frass or with black linear frass, broadens rather abruptly into gallery with central line of black frass; exit-hole on upperside, a semicircular slit. Cocoon: reddish brown. Occupied mines have been collected on 25 June, 1 July, 13 July and 3 August.

#### Diagnosis.

The male is superficially similar to that of *Pectinivalva (Casanovula) minotaurus*, but differs in its much less strongly expanded antennae. The male of *Pectinivalva (Casanovula) minotaurus* also differs in having shell-like androconial scales on the upperside of the abdomen, visible on dissection, and a more distinctly H-shaped vinculum (Figs 46, 62). The female of *Pectinivalva brevipalpa* is also very similiar to that of *minotaurus* but can be distinguished on dissection by the presence of the indistinct sclerites ½ way down the corpus bursae.

#### Distribution.

New South Wales.

#### Derivation.

The specific name is derived from the Latin *brevis* (short) and *palpus* (the sensitive palm of the hand: hence, in zoology, a palp) and refers to the reduced, 2-segmented labial palpi of the adult male. It is an adjective.

### Pectinivalva (Casanovula) minotaurus

Hoare sp. n.

urn:lsid:zoobank.org:act:72FAEDB0-F837-4C78-90E3-3ABB6F805085

http://species-id.net/wiki/Pectinivalva_minotaurus

#### Material examined.

Holotype. ♂, 27.36S 151.59E, Leslie St., Toowoomba, Qld, emg. 19.ii.1996, *Lophostemon confertus*, R.J.B. Hoare, I.F.B. Common. Paratypes. 2♂, 6♀, same data as holotype, emg. 2.-27.ii., 1.iii.1996, slide 11325 (anic); 11♂, 17♀, same locality, emg. 2.i.-2.ii.2001, *Lophostemon confertus*, R.J.B. Hoare, C. van den Berg, genitalia slides CvdB110, EvN 3539, 3547 (rmnh); 3♀, 27.33S, 151.59E, Prince Henry Heights, Toowoomba, Queensland, emg. 15, 18.ii.1986, *Lophostemon confertus*, I.F.B. Common, slide 10209 (anic); 1♂, 1♀, Brisbane, Queensland, emg. 30.xii.1957, *Lophostemon suaveolens*, I.F.B. Common, slides 11507, 11582 (anic); 1♂, Goodna, [Queensland], 8.iv.1906, [A.J. Turner], slide 11506 (anic).

#### Description.

Male (Fig. 8). Wingspan 4.7–5.5 mm. Head capsule (Figs 27–30): labial palpi 3-segmented; segment 2 reduced, maxillary palpi with ratio of segments from base approximately 0.3: 0.8: 0.6: 1.7: 1.0; interocular index 0.57; vertex with a pair of sclerotized crests. Frontal tuft ferruginous; collar ferruginous; eyecaps white, posteriorly leaden; antennae with flagellomeres in basal ½ greatly dilated and flattened, tapering beyond this, shining lead-grey, yellowish beneath, ca. 43–48 segments. Thorax and tegulae dark fuscous with purplish reflections. Forewing to ½ dark fuscous with bluish and purplish reflections; beyond this dark fuscous with bronzy reflections; a shining pale golden fascia at 2/3, apex of wing at base of cilia with purplish reflections; cilia grey beyond a line of fuscous-tipped scales, pale brownish around apex. Hindwing grey, unmodified; cilia grey. Abdomen with T2–3 shining brassy golden, remaining tergites shining dark leaden with green and violet reflections; T4 laterally with contiguous groups of androconial scales of two types: inner scales scallop-shaped, finely ridged; outer scales calyx-shaped, coarsely ribbed; T5 with similar area of androconia consisting entirely of scallop-shaped scales (Figs 37, 38), these showing as velvet black crescents on abdomen *in situ*.

Female (Fig. 9). Wingspan 4.7–5.8 mm. Similar to male, but antennae not dilated at base, ca 21–24 segments; abdomen entirely leaden with brassy reflections.

Male genitalia (Figs 46–48, 62, 63). Capsule ca. 360–375 μm. Anterior extension of vinculum reduced to curved lateral struts, i.e. vinculum anteriorly H-shaped. Uncus subtriangular, bilobed, with a compact tuft of setae arising from dorsal side of each lobe near tip. Gnathos with elongate central element and short lateral arms. Valva (Fig. 47) ca. 235 μm, squarish, caudal margin very straight; pectinifer consisting of ca. 29 narrow elements. Transtilla absent. Aedeagus (Figs 48, 63) 545 μm, with single broad, blunt apical process. Vesica with numerous close-set spine-like cornuti in several groups.

Female genitalia (Fig. 75, 86–88). Total length 800–880 μm. T9 with ca 9 setae on each side. Apophyses anteriores reduced to rounded stubs; apophyses posteriores narrow, much longer than anteriores. Lateral sclerotizations of vestibulum narrow, bent inwards, tips squared off. Ductus spermathecae with 1 ½ convolutions. Posterior part of corpus bursae very convoluted; anterior part with many coarse pectinations in right half; left half with a few fine pectinations only; no further sclerotizations in corpus.

Larva. Green. Head (Fig. 106) parallel-sided; length of head ca. 250 μm; width ca. 215 μm. Thorax: prothoracic sternite as in Fig. 111. Chaetotaxy as described for subgenus; T2 with 11 pairs of setae (L3 present), A10 probably with 3 pairs (but 1 pair possibly lost in slide examined). Anal rods distinctly forked posteriorly.

#### Biology.

Host plants: *Lophostemon confertus* (R.Br.) Peter G.Wilson & J.T.Waterh.and *Lophostemon suaveolens* (Sol. ex Gaertn.) Peter G.Wilson & J.T.Waterh. (Myrtaceae). Egg: invariably on upperside of leaf. Mine (Fig. 118): commences as very long narrow gallery with black linear frass, leaving narrow clear margins, broadens rather abruptly into an irregular wide gallery or elongate blotch, sometimes with gallery parts, with central line of black frass or in the case of the blotch, frass concentrated on one or both sides; exit-hole on underside, an almost circular hole. Cocoon (Fig. 125): dark reddish brown. Occupied mines have been collected on 6 and 17 July and 15 August. A male pupa (pharate adult) is shown in Figs 126, 127.

#### Diagnosis.

Very similar externally to *Pectinivalva (Casanovula) brevipalpa* in both sexes; diagnostic characters are listed under that species.

#### Distribution.

Southern Queensland.

#### DNA barcode.

RMNH.INS.23539, Genbank KC292478 and RMNH.INS.23547, Genbank KC292477, both identical.

#### Derivation.

The species is named after the famous beast of Greek mythology, the Minotaur. The name (a noun in apposition) refers to the extraordinarily expanded and flattened male antennae, which are likened to the Minotaur’s horns.

#### Remarks.

The antennae of the male are the most strongly modified of any known species of Nepticulidae. Although many male-specific head structures in other insects are utilized in male-male competitive interactions over mates (e.g. the lateral cephalic projections of *Phytalmia* spp, Tephritidae (Moulds 1977)), such direct competition is unknown in Lepidoptera, and the antennae of *minotaurus* are more likely to function in close-range courtship, along with the androconial scales on the male abdomen. Similar widened flagellomeres are known from the genus *Thisizima* Walker, 1864 in Tineidae (Yang et al. 2012). The androconial scales are also remarkable, two distinct types being present in contiguous patches on the abdominal dorsum.

### Subgenus Menurella

Hoare subgen. n.

#### Type species.

*Pectinivalva (Menurella) scotodes* sp. n.

#### Description.

Adults. Head capsule (Figs 21, 22): labial palpi 3-segmented; interocular index 0.49–0.84. Antennae in male occasionally broadened in middle, or with pedicel and segment 1 of flagellum modified as in Fig. 22. Collar consisting of piliform scales (lamellate scales in one undescribed species). Wingspan3.2–7.0 mm. Thorax and forewing usually unicolorous greyish to fuscous, or with transverse pale fascia or opposite pale spots on costa and tornus at 2/3, occasionally yellowish with dark markings. Costa of forewing in male sometimes with a tuft of very narrow stiff scales towards base. Hindwing of male occasionally expanded at base; androconial pocket often present. Underside of forewing in male sometimes with androconia. Wing venation (Figs 34–36): R2+3 in forewing absent. Abdomen: S2a with or without spines. Legs: fore-tibia in male of those species with androconial pocket usually thickened above with blackish scales.

Male genitalia (Figs 49–57, 64–72). Vinculum with lateral arms often conspicuously forked apically; the lower branchsupporting the uncus and the upper branch the tegumen. Uncus apically bifid. Valva (Figs 50, 53, 56) variable in shape; pectinifer with ca. 9–34 (usually fewer than 20) peg-like, spine-like or broad tooth-like elements, or reduced to a thickening along caudal edge of valva. Transverse bar of transtilla absent (present in *Pectinivalva (Menurella)* 119). Aedeagus (Figs 51, 54, 57, 65, 66, 69, 72): cathrema associated with the apex of a long tubular sclerotization.

**Figures 49–57.**
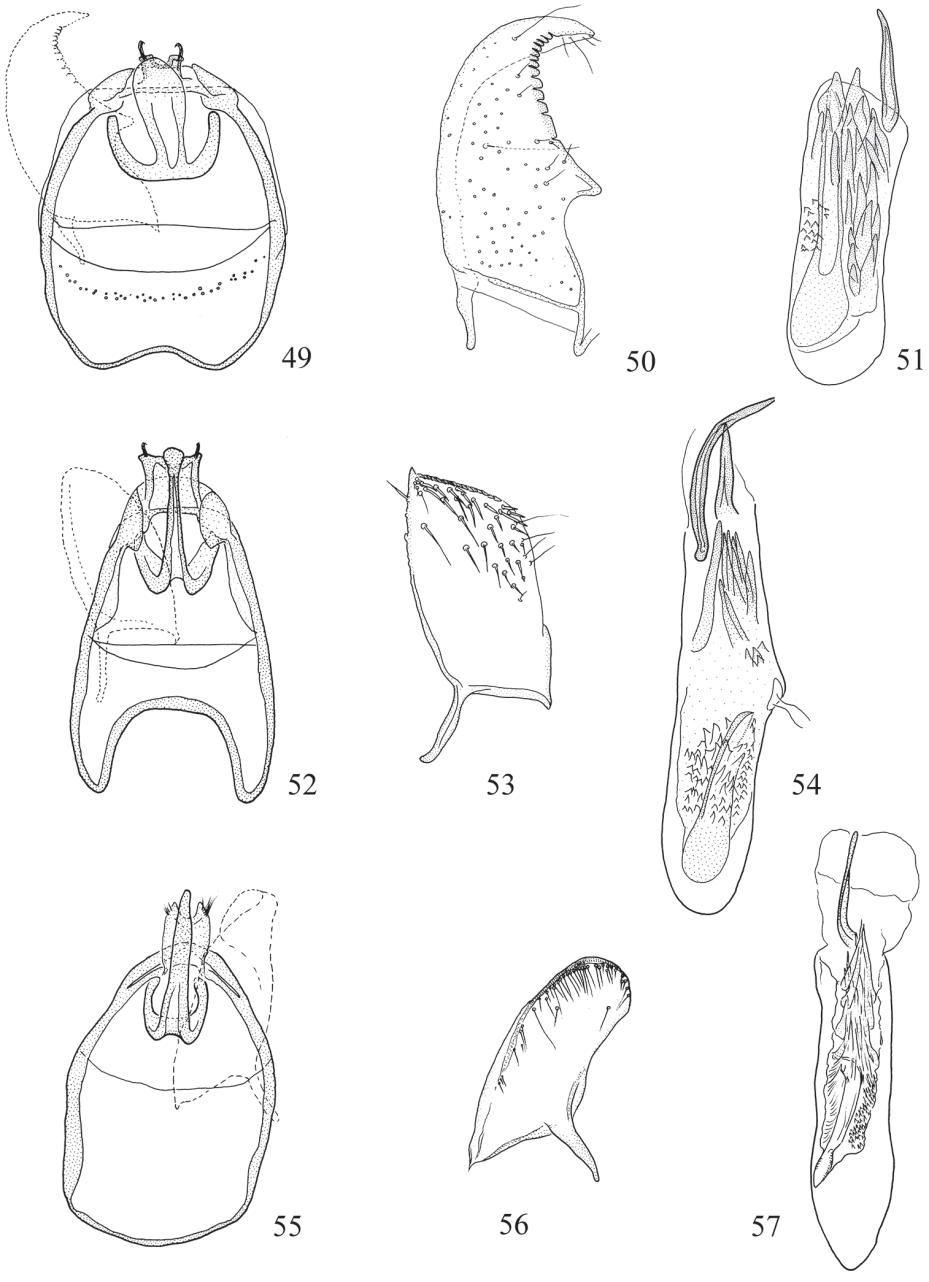
*Pectinivalva (Menurella)* spp., male genitalia, ventral view. **49–51** *Pectinivalva (Menurella) scotodes*: **49** genital capsule **50** left valva **51** aedeagus **52–54** *Pectinivalva (Menurella) acmenae*: **52** genital capsule **53** left valva **54 **aedeagus **55–57** *Pectinivalva (Menurella) quintiniae*: **55** genital capsule **56** left valva **57** aedeagus.

**Figures 58–66.**
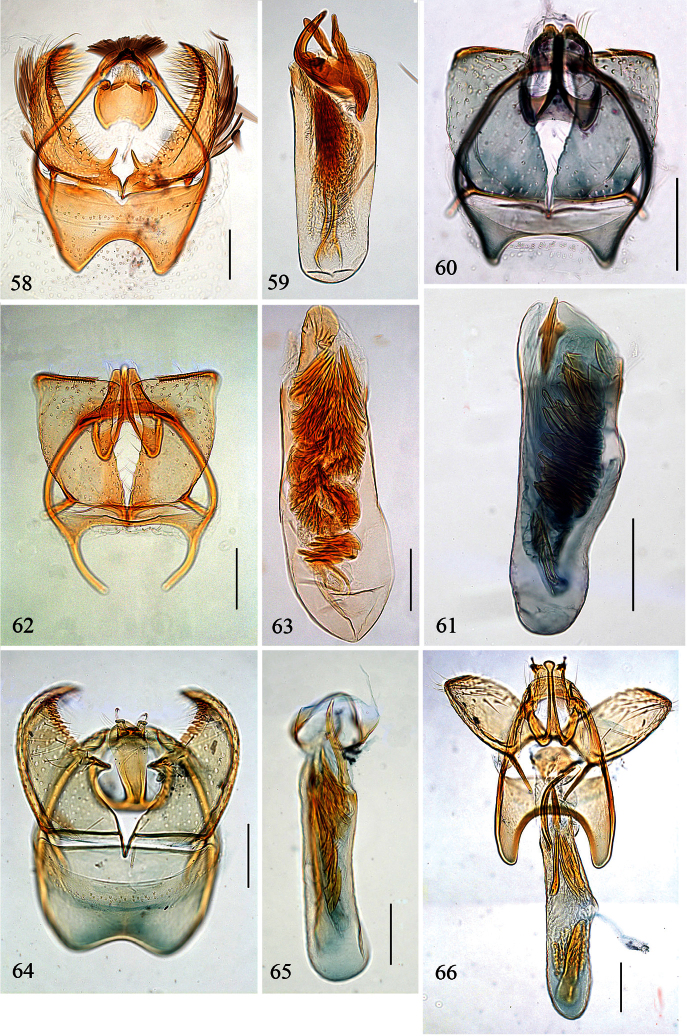
*Pectinivalva* spp., male genitalia, ventral view. **58, 59** *Pectinivalva (Pectinivalva) mystaconota*, paratype, slide EJvN 4106: **58** genital capsule **59** aedeagus **60, 61** *Pectinivalva (Casanovula) brevipalpa*, holotype, slide ANIC12111: **60** genital capsule **61** aedeagus **62, 63** *Pectinivalva (Casanovula) minotaurus*, paratype, slide CvdB110: **62** genital capsule **63** aedeagus **64, 65** *Pectinivalva (Menurella) scotodes*, paratype, slide ANIC11262: **64** genital capsule **65** aedeagus **66** *Pectinivalva (Menurella) acmenae*, paratype, slide ANIC10213 **66** genital capsule with aedeagus almost *in situ*. Scales 100 μm (59 same scale as 58).

**Figures 67–72.**
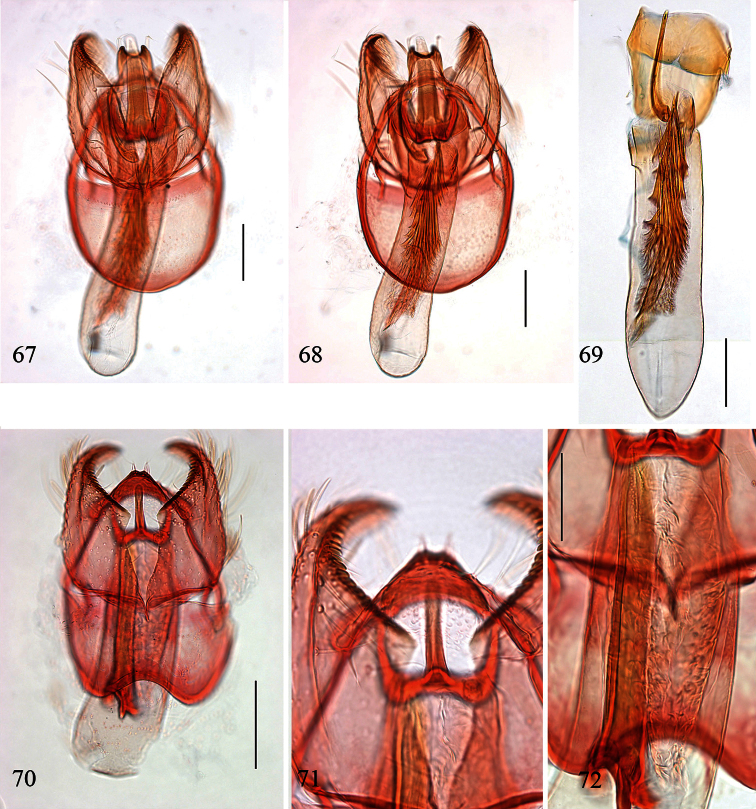
*Pectinivalva (Menurella)* spp., male genitalia, ventral view. **67–69** *Pectinivalva (Menurella) quintiniae*, holotype, slide ANIC18720 and paratype, slide EJvN3736 (69): **67**, **68** genitalia with aedeagus *in situ* **69** aedeagus **70–72** *Pectinivalva (Menurella) tribulatrix*, holotype, slide ANIC18721: **70** genitalia with aedeagus *in situ* **71 **close-up of gnathos and pectinifer **72** close-up of aedeagus showing tubular sclerite associated with cathrema. Scales 100 μm, 50 μm (71, 72).

Female genitalia (Figs 76–79, 89–103). Lateral sclerites of vestibulum present: forked or thickened. Accessory sac absent. Corpus bursae with extent of pectinations more or less reduced; signum either a small weakly toothed band (*Pectinivalva (Menurella) acmenae*, *Pectinivalva (Menurella) xenadelpha* and *Pectinivalva (Menurella) quintiniae*) or 2 concentric ovals of fence-like marks.

Larva. Head (Figs 107, 108) always more or less cordate, never pyriform. Chaetotaxy: T2 with 10 or 11 pairs of setae (D1 usually present; L3 present or absent). Otherwise not distinguished from that of *Pectinivalva (Casanovula)*.

#### Biology.

Host-plants: *Syzygium* R.Br. ex Gaertn.species (formerly in *Acmena*), *Leptospermum* Forst. et f. spp., *Angophora* Cav. spp., *Corymbia maculata* (Hook.) K.D. Hill & L.A.S. Johnson, *Rhodomyrtus macrocarpa* Benth., *Eucalyptus* spp., probably other Myrtaceae, and with one species on Paracryphiaceae (*Quintinia*). Mine (Figs 119–124): usually a narrow gallery more or less filled with frass, occasionally a very short gallery leading to and enveloped by a blotch; exit-hole usually a small semicircular hole.

#### Diagnosis.

See Table 1.

#### Distribution.

Australia (known from all states and territories), Indonesia (Borneo: Kalimantan).

#### Derivation.

The subgeneric name is the diminutive of *Menura*, the genus to which the lyre-bird belongs. It stems from a fancied resemblance between the uncus in some species of the group and the tail of the male lyre-bird. It should be treated as feminine.

#### Included species.

In addition to eleven described and new species, also approximately 70 undescribed species in the anic, of which the following, cited by their anic rearing numbers, have been studied in detail for the current work: *Pectinivalva (Menurella)* 2; *Pectinivalva (Menurella)* 91; *Pectinivalva (Menurella)* 119.

#### Discussion.

*Menurella* is the most diverse of the three subgenera of *Pectinivalva*, in terms of numbers of species, morphology and host-plant choice. Below we describe the type species *Pectinivalva (Menurella) scotodes*, three morphologically unusual rainforest species *Pectinivalvaacmenae*, *Pectinivalva xenadelpha*, *Pectinivalva tribulatrix*, and *Pectinivalva quintiniae* with unusual morphology and host-plant.

*Pectinivalva (Menurella)* is equivalent to the *Pectinivalva funeralis* group of Hoare et al. (1997).

### Pectinivalva (Menurella) scotodes

Hoare sp. n.

urn:lsid:zoobank.org:act:62D9A762-334E-4FDE-A191-0282BED3FC18

http://species-id.net/wiki/Pectinivalva_scotodes

#### Material examined.

Holotype. ♂, 27.36S, 151.59E, Leslie St., Toowoomba, Queensland, emg. 8.x.1995, *Eucalyptus pilularis*, R.J.B. Hoare, I.F.B. Common. Paratypes. 6♂, 4♀, same data as holotype, emg. 3–10.x., 9.xi., 13.xii.1995, slides 11330, 12066 (anic); 5♂, 4♀, 27.36S, 151.59E, J.E. Duggan Park, Leslie St., Toowoomba, Queensland, 6.vii.2000, emg. 11.viii.-27.ix.2000, *Eucalyptus pilularis*, R.J.B. Hoare, C. van den Berg, bred in NL, slide EJvN3548 (rmnh); 4♂, 2♀, McAfee’s Lookout, Brisbane Forest Park, Queensland, emg. 1–10.x.1995, *Eucalyptus carnea*, R.J.B. Hoare, slides 11262, 11263 (anic).4♂, 3♀, Lisarow, N.S.W., emg. ix.1954, ix.-x.1955, x.1956, *Eucalyptus acmenoides*,K.M. Moore; 1♀, Mollymook, N.S.W., 21.xii.1996, emg. 28.i.1997, *Eucalyptus pilularis*, R.J.B. Hoare.

#### Description.

Male (Fig. 10). Wingspan 5.2–5.7 mm. Head capsule (Figs 21, 22): labial palpi distinctly shorter than galeae; maxillary palpi with ratio of segments from base approximately 0.4: 0.3: 0.4: 1.4: 1.0; interocular index 0.69; scape slightly expanded posteriorly into a setose ‘bump’; 1st 2 flagellar segments of antenna fused and narrower than remaining segments so that base of flagellum appears slightly invaginated posteriorly. Frontal tuft black, collar white; eyecaps white, black-bordered posteriorly beneath; antennae shining grey, ca. 42 segments. Thorax and forewing entirely blackish brown; a row of long blackish androconial scales projecting from dorsum; cilia shining dark brown, cilia-line indistinct. Hindwing rather broad, dark brown, with a small narrow androconial pocket basally; cilia shining blackish. Underside: forewing dark brown, costa black with a knob of black granular scales at base forming retinaculum; hindwing dark brown with blackish lamellate scales along basal ½ of costa. Wing venation as in Fig. 35. Legs: fore-tibia thickened above with blackish scales. Abdomen shining blackish.

Female (Fig. 11). Wingspan 5.0–5.2 mm. Head: frontal tuft brownish, collar white; eyecaps shining white, unmodified, antennae shining grey, ca. 24 segments, basal flagellar segments unmodified. Thorax and forewing paler than in male, yellowish overlain more or less extensively with brownish fuscous scales, leaving following markings yellow: a diffuse streak just beneath costa reaching ½ way along wing and diffuse opposite spots on costa and tornus at 2/3, cilia grey with moderately distinct cilia-line. Hindwing narrower than in male, grey; cilia grey. Underside: forewing shining dark brown; hindwing shining grey. Wing venation as in Fig. 34. Legs unmodified. Abdomen shining dark grey, paler beneath.

Male genitalia (Figs 49–51, 64, 65). Capsule ca. 370 μm long. Vinculum with slight anterior excavation; lateral arms inconspicuously forked apically, the caudal bifurcations from each side uniting to form straight bar along base of tegumen. Tegumen narrow, lateral corners produced anteriorly into distinct ‘shoulders’. Uncus small, boat-shaped. Gnathos central element long, spatulate. Valva (Fig. 50) ca. 245 μm long, reaching well beyond tegumen, strongly curved; medial edge smoothly excavated and ending in triangular projection; pectinifer consisting of 12 broad, blunt elements; dorsal surface towards apex with long setae. Juxta consisting of paired plate-like sclerites. Aedeagus (Fig. 51, 65) ca. 455 μm long; a spine-like process projecting from apex on right in ventral view; vesica with ca. 20 rather large cornuti, the 2 apical ones with very broad bases; sclerotized tube supporting cathrema very long, 2/3 length of aedeagus.

Female genitalia (Fig. 76, 89–91). Total length ca. 640 μm. T9 with ca. 10–11 setae on each side. Apophyses posteriores slightly longer than anteriores; apophyses anteriores curved inwards. Segment 7 produced laterally into 2 small evaginations either side of apophyses anteriores. Lateral sclerites of vestibulum broad, their apices associated with a pair of roughened irregular sclerotizations in centre of vestibulum. Ductus bursae strongly folded. Ductus spermathecae with ca. 3–4 poorly defined convolutions. Corpus bursae rounded; a field of concentrically arranged pectinations in posterior ½ on one side; signum a pair of concentric ovals of fence-like spines.

Larva. Appearing translucent whitish or yellowish in mine, becoming dull purplish white on vacating. Head as in Fig. 107; length of head ca. 345 μm; width ca. 270 μm. Thorax: prothoracic sternite (Fig. 112) narrow, I-shaped. Chaetotaxy and spinosity: T2 with 10 pairs of setae (L3 absent); otherwise as described for subgenus *Casanovula*. Anal rods slightly forked posteriorly.

**Figures 73–76.**
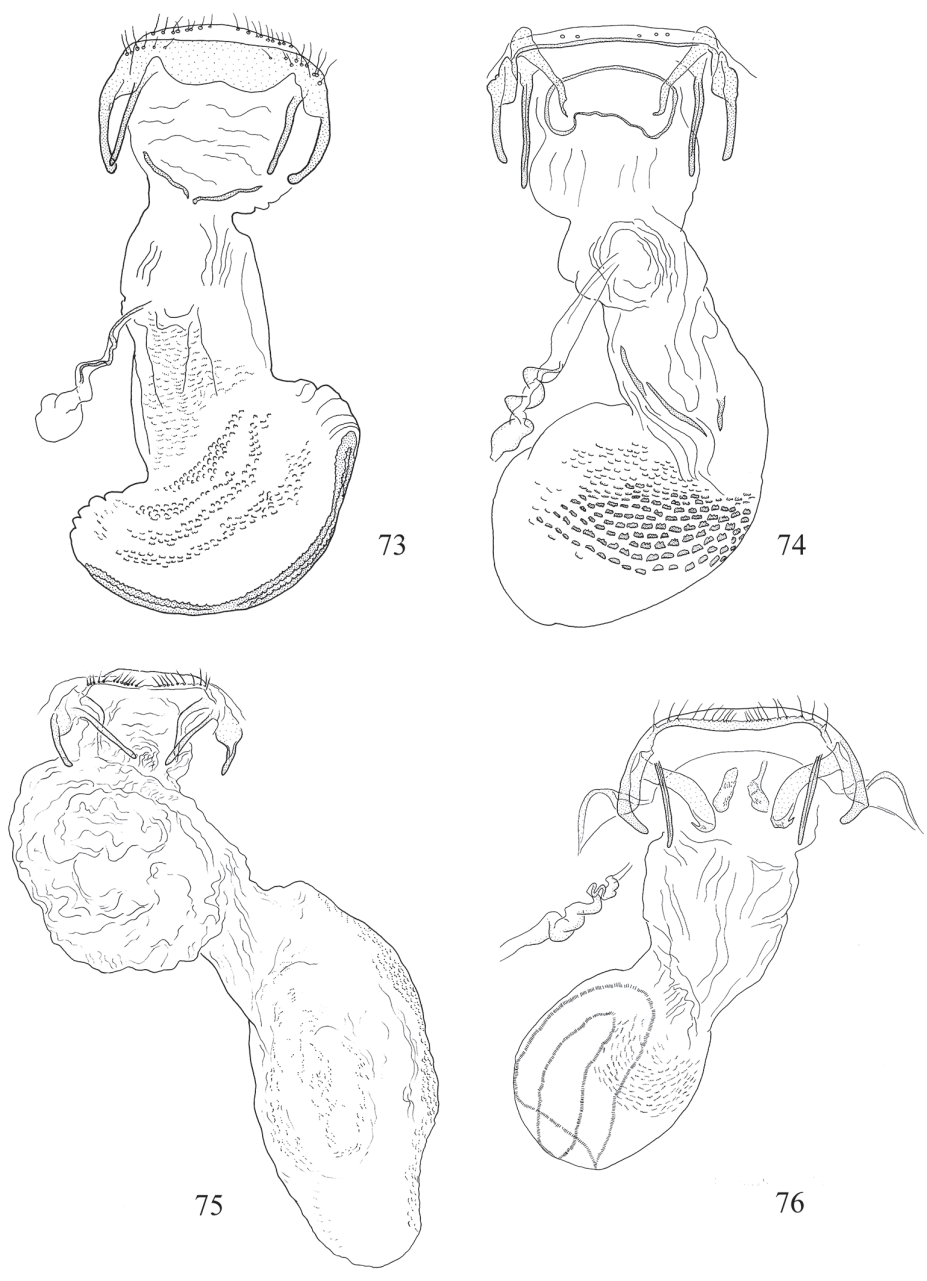
*Pectinivalva* spp., female genitalia, ventral view. **73** *Pectinivalva (Pectinivalva) mystaconota* **74** *Pectinivalva (Casanovula) brevipalpa* **75** *Pectinivalva (Casanovula) minotaurus* **76** *Pectinivalva (Menurella) scotodes*.

**Figures 77–79.**
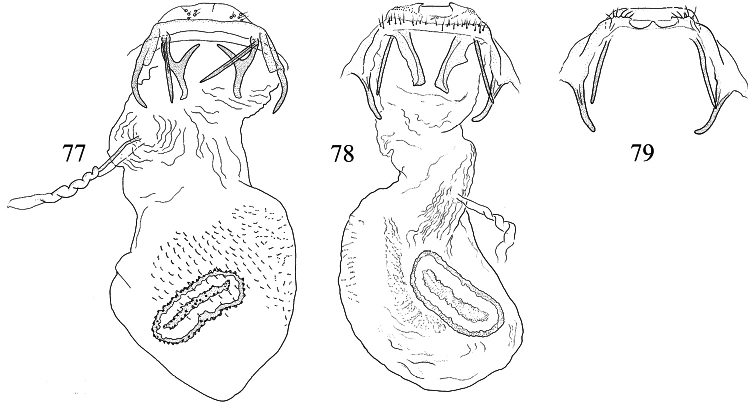
*Pectinivalva (Menurella)* spp., female genitalia, ventral view. **77** *Pectinivalva (Menurella) acmenae* **78** *Pectinivalva (Menurella) quintiniae* **79** *Pectinivalva (Menurella) quintiniae*, apophyses and papillae anales.

**Figures 80–88.**
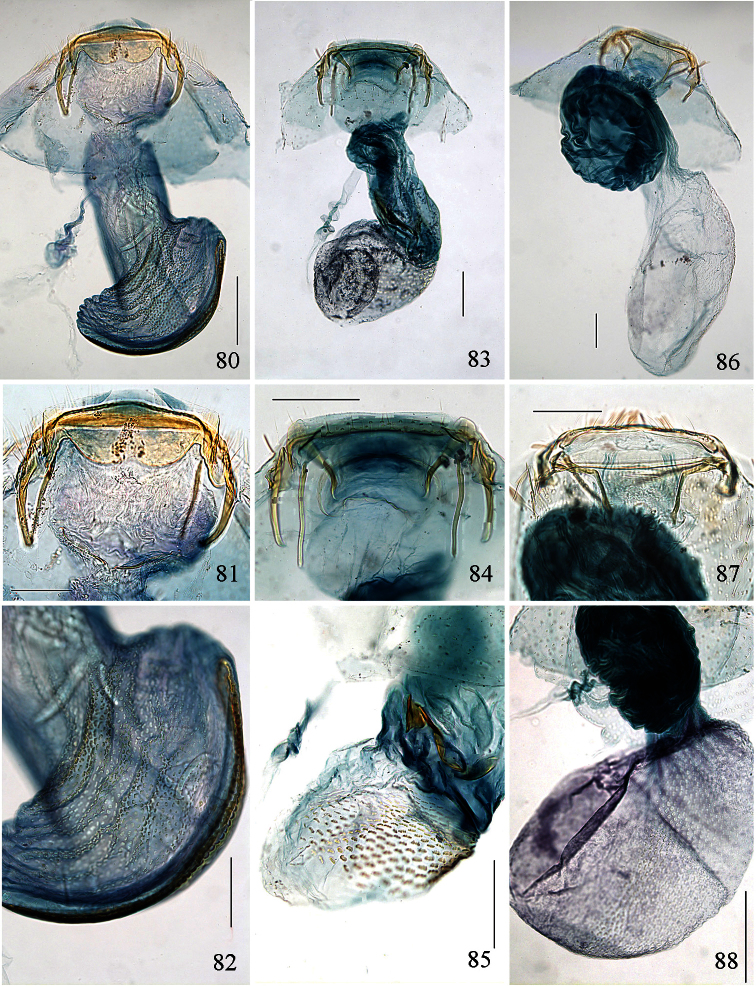
*Pectinivalva* spp., female genitalia, ventral view. **80–82** *Pectinivalva (Pectinivalva) mystaconota*, paratype, slide ANIC10161 **83–85** *Pectinivalva (Casanovula) brevipalpa*, paratypes, slides ANIC11328, ANIC11238 (85) **86–88** *Pectinivalva (Casanovula) minotaurus*, paratypes, slides ANIC11327 (86), ANIC10209. Scales 100 μm, 200 μm (80, 88).

**Figures 89–97.**
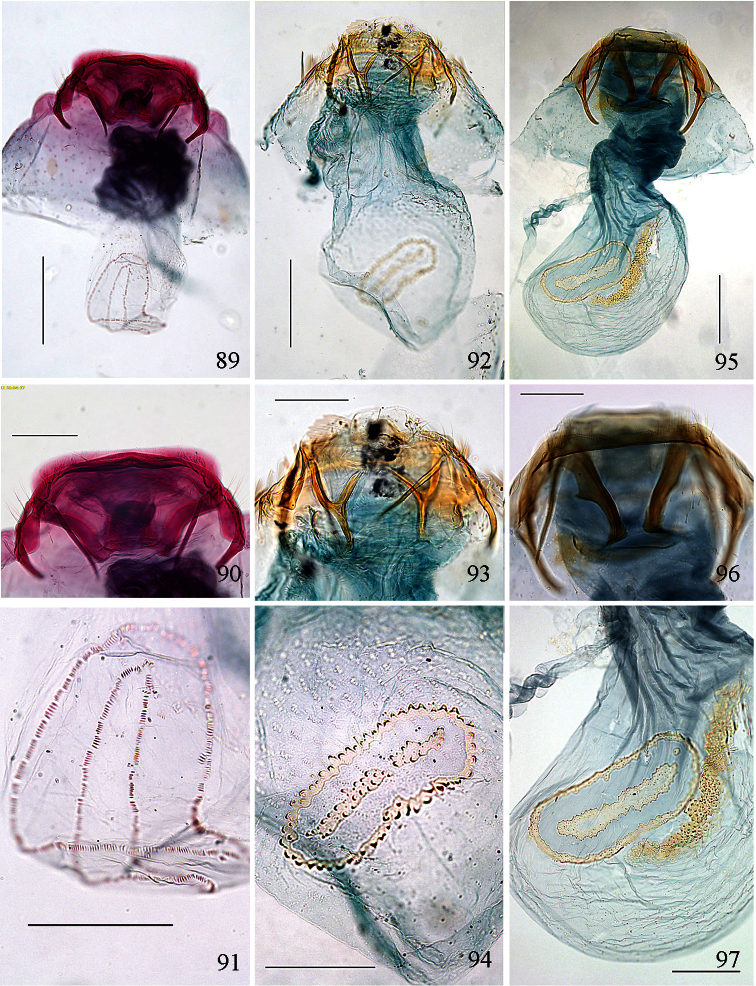
*Pectinivalva(Menurella)* spp., female genitalia, ventral view. **89–91** *Pectinivalva (Menurella) scotodes*, paratype, slideANIC11263 **92–94** *Pectinivalva (Menurella) acmenae*, paratype, slideANIC11242 **95–97** *Pectinivalva (Menurella) quintiniae*, paratype, slide EJvN3961. Scales 200 μm (89, 92, 95), 100 μm.

#### Biology.

Host plants: *Eucalyptus pilularis* Smith, *Enteucha carnea* R. Baker, *Enteucha acmenoides* Schauer and probably *Enteucha saligna* Smith (see below) (Myrtaceae). Egg: on upperside of leaf. Mine (Fig. 119): commences as a tight spiral around the egg, causing a raised red-brown spot on the leaf about 3–5 mm in diameter; later broadens into a more or less contorted linear gallery with black frass leaving narrow clear margins; exit-hole on leaf underside, a crescentic hole. Often several mines to a leaf. Cocoon: reddish brown. Occupied mines were collected on 17 and 20 July 1995, and 21 Dec 1996, and have also been recorded in January, March, April, June and August (Moore 1966).

#### Diagnosis.

The male of *Pectinivalva (Menurella) scotodes* resembles those of *Pectinivalva (Menurella) funeralis* (Meyrick), *Pectinivalva (Menurella) libera* (Meyrick) and *Pectinivalva (Menurella)* 119. It can be distinguished from all of these by its black head-tuft. The brown and yellow wing pattern of the female is distinctive amongst known species (although the females of *Pectinivalva (Menurella) funeralis* and *Pectinivalva (Menurella) libera* are unknown). The unusual larval mine of *Pectinivalva (Menurella) scotodes* appears to be diagnostic.

#### Distribution.

N.S.W, Southern Queensland.

#### DNA barcode.

RMNH.INS.23548, Genbank KC292483.

#### Derivation.

The specific name (an adjective) is derived from the Greek *skotodes*, meaning either ‘dark’ or ‘dizzy’. It refers both to the blackish coloration of the adult male moth, and to the habit of the young larva, which mines in tight circles.

#### Remarks.

The two female paratypes reared from mines on *Eucalyptus carnea* collected near Brisbane have a sparser scattering of brown scales on the yellow ground colour than the females reared from *Enteucha pilularis*. However, no other differences have been observed between specimens from these two host-plants, and the mines also appear to be identical.

This species was first collected by K.M. Moore, who described and illustrated the mine (Moore 1966: figs 15, 15A). There are specimens in the anic (here designated paratypes) reared by him in the 1950’s. The host-plant is indicated on the labels only by a rearing number; mines from his herbarium with the corresponding number are all in leaves of *Eucalyptus acmenoides*. He also recorded mines on *Enteucha saligna*, but no specimens reared from this host-plant have been located. Moore referred to this species as ‘*Nepticula* sp. 3’ and regarded it as related to *Nepticula gilva* Meyrick. He was probably misled by the wing-pattern of the female of *Pectinivalva (Menurella) scotodes*, which bears some resemblance to that of *Pectinivalva (Pectinivalva) gilva*: he would not have seen the type specimen of *Pectinivalva (Pectinivalva) gilva* in the BMNH. The two species are not closely related and belong to different subgenera of *Pectinivalva*.

### Pectinivalva (Menurella) acmenae

Hoare sp. n.

urn:lsid:zoobank.org:act:6720E8F8-1B68-45B0-B6EE-A94F9D8F0ADA

http://species-id.net/wiki/Pectinivalva_acmenae

#### Material examined.

Holotype. ♂, 35.37S, 150.16E, 1 km SE of East Lynne, Kioloa State Forest, N.S.W., *Acmena smithii*, emg. 12.x.1995, R.J.B. Hoare. Paratypes. 3♂, 3♀, same data as holotype, emg. 10–21.x.1995; 2♂, 36.19S, 150.03E, Mt Dromedary, N.S.W., emg. 22, 24.x.1995, R.J.B. Hoare, E.S. Nielsen and M.J. Matthews, genitalia slides 10213, 11242 (anic); 2♂, 28.42S,153.37E, Broken Head NR, N.S.W., 13.vii.2000, emg. 15–18.viii.2000, *Acmena smithii*, R.J.B. Hoare, C. van den Berg, bred in NL, slide EJvN3541 (rmnh).

#### Description.

Male (Fig. 12). Wingspan 4.5–5.5 mm. Head: frontal tuft ferruginous; collar inconspicuous, consisting of white, grey-tipped scales; eyecaps anteriorly white, posteriorly shining grey with bluish reflections; antennae shining dark grey, whitish beneath, ca. 35 segments. Thorax, tegulae and forewing uniform shining dark grey with strong blue reflections; an inconspicuous tornal spot consisting of a few white scales; cilia dark grey. Hindwing unmodified, pale grey; cilia pale grey. Underside: forewing grey with faint brassy reflections; hindwing grey. Abdomen shining dark grey; anal tuft inconspicuous, dark grey.

Female (Fig. 13). Wingspan 5.2–5.6 mm. Similar to male, but antenna with 23–25 segments, and forewing rather broader. Wing venation as in Fig. 36. Abdominal tip not as broad and ‘square’ as in females of other *Pectinivalva* spp.

Male genitalia(Figs 52–54, 66). Capsule ca. 425 μm long, forming a narrow triangle. Anterior edge of vinculum excavated in a half-oblong. Tegumen rounded, with ventral extensions on each side overlapping lateral arms of gnathos. Uncus rectangular, bilobed, lobes slightly produced, with 3 setae on each. Gnathos central element long, reaching just beyond uncus, ending in small swelling. Valva (Fig. 53) ca. 210 μm long, squarish, more rounded caudally and produced into a short point at exterior corner of apex; apical ½ with numerous spine-like setae on dorsal surface; pectinifer consisting of ca. 18 spine-like elements. Long sublateral processes present. Juxta a weak subcircular plate. Aedeagus (Figs 54, 66) ca. 510 μm long, a curved spine arising towards apex on left, a shorter spine to right of this one, a third spine in line with second and anterior to it. Vesica basally with cathrema surrounded by a field of many broad, short cornuti; a separate field of ca. 9 long narrow cornuti above opening of ejaculatory duct.

Female genitalia(Fig. 77, 92–94). Total length ca. 760 μm. T9 prominent, with a group of 5–6 setae on each side. Apophyses anteriores rather narrow, curved inwards; apophyses posteriores narrow, straight, approximately equal in length to anteriores. Lateral sclerotizations of vestibulum strongly developed, forked, the bifurcations diverging widely, anterior pair blunt, posterior pair pointed. Ductus spermathecae with 4½ convolutions. Posterior part of corpus broad, folded, without markings; anterior part rounded, with rows of inconspicuous pectinations; signum consisting of broken linear sclerotization surrounded by oval sclerotized ring with blunt dentitions.

Larva. Green. Length of head ca. 440 μm; width ca. 350 μm. Thorax: prothoracic sternite in shape of Y with expanded base (Fig. 113); an additional small roundish sclerite on each side of this and antero-dorsal to SV and V group of setae. Chaetotaxy and spinosity: T2 with 11 pairs of setae (L3 present); otherwise as described for subgenus *Casanovula*. Anal rods distinctly forked posteriorly.

#### Biology.

Host plant: *Syzygium smithii* (Poir.) Nied. (Myrtaceae) (formerly *Acmena smithii*), common lilly pilly. Egg: almost invariably on upperside, usually near leaf margin. Mine (Fig. 120): a long, very narrow contorted gallery, filled with brown frass apart from irregular crenulations along mine edge; exit-hole on underside, a semicircular hole. Cocoon: reddish brown. Occupied mines were collected on 30 July and 3 August.

#### Diagnosis.

This is the one of three known species of *Pectinivalva* in which the forewings have a bluish lustre but no transverse fascia. The others are *Pectinivalva xenadelpha* and *Pectinivalva quintiniae*, both described and diagnosed below. There is an undescribed Australian species of *Stigmella* which sometimes occurs together with *Pectinivalva (Menurella) acmenae*, and in which the forewings are similarly unicolorous dark blue; however, the *Stigmella* species is distinctly larger (wingspan 6–8 mm), and has a collar consisting of white lamellate scales; its larva is a leaf-miner on *Baloghia inophylla* (G. Forster) P. Green (Euphorbiaceae).

#### Distribution.

New South Wales. Vacated mines probably of this species were seen abundantly along the coast near Manley, Sydney.

#### DNA barcode.

RMNH.INS.23541, Genbank KC292474.

#### Derivation.

The specific name is derived from the former host-plant genus, and is a noun in the genitive. Because the moth is referred to under this manuscript name in the first author’s unpublished thesis, we have chosen to retain it for consistency, in spite of the change in classification of the host-plant.

#### Remarks.

The host-plant genus of *Pectinivalva (Menurella) acmenae*, *Syzygium*, is not closely related to other myrtaceous hosts from which *Pectinivalva* species have been reared in Australia, and belongs to the tribe Syzygieae (Wilson et al. 2005, Biffin et al. 2006). Vacated mines on *Syzygium ingens* (F.Muell. ex C.Moore) Craven & Biffin (= *Acmena brachyandra*) in Lamington National Park have tentatively been identified as this species (Appendix 2 and online Appendix 3).

### Pectinivalva (Menurella) xenadelpha

Van Nieukerken & Hoare sp. n.

urn:lsid:zoobank.org:act:3F7C0ED7-9A5A-4A4D-8D5D-FFB1DE384D85

http://species-id.net/wiki/Pectinivalva_xenadelpha

#### Material examined.

Holotype. ♀, INDONESIA (Kalim. Timur), Pasir distr.: Gunung Lumut Prot. For., Gunung Lumut, ridge SW of summit, 18–20.xi.2005, 50M LD864441, 950 m, leafmines, undisturbed *Acmena* dominated low forest, on *Acmena acuminatissima* (Blume) Merr. & L.M. Perry, emg. 15.xii.2005, RMNH/EvN no 2005185–1, E.J. van Nieukerken, genitalia slide EvN 3738 (mzb).

**Additional material:** leafmines, larvae, same locality (rmnh).

#### Description.

Male. Unknown.

Female (Fig. 14, 129). Wingspan 4.0 mm. Head: frontal tuft pale ferruginous; collar inconspicuous, white; eyecaps anteriorly white, posteriorly shining grey with bluish reflections; antennae shining dark grey, 24 segments. Thorax, tegulae and forewing uniform shining dark grey with weak blue reflections; cilia dark grey. Hindwing pale grey; cilia pale grey. Underside: forewing grey with faint brassy reflections; hindwing grey. Abdomen shining dark grey, abdominal tip as in *acmenae*.

**Figures 98–103.**
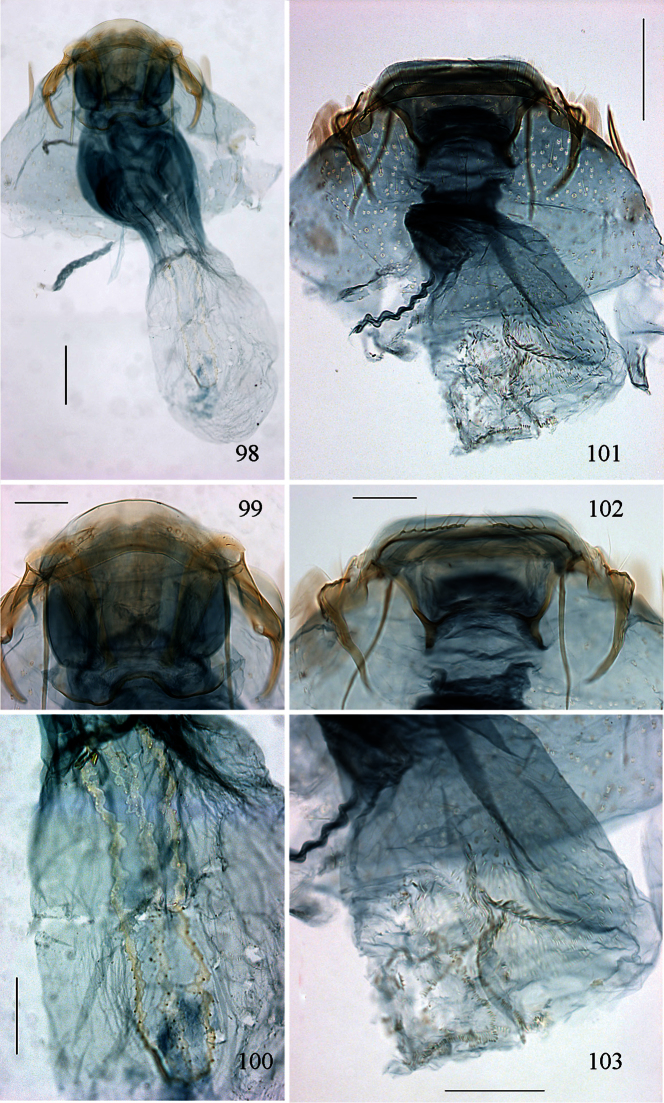
*Pectinivalva (Menurella)* spp., female genitalia, ventral view. **98–100** *Pectinivalva (Menurella) xenadelpha*, holotype, slide EJvN3738 **101–103** *Pectinivalva (Menurella) tribulatrix*, paratype, slide EJvN3963. Scales 100 μm, 50 μm (99, 100, 102).

Female genitalia (Figs 98–100). Total length ca. 770 μm. T9 prominent, with a group of 5 setae on each side. Apophyses anteriores rather narrow, curved inwards; apophyses posteriores narrow, straight, longer than anteriores. Lateral sclerotizations of vestibulum strongly developed, but not forked. Ductus spermathecae with ca 6 close set convolutions. Posterior part of corpus normal, slightly folded, without markings; anterior part rounded, with rows of inconspicuous pectinations; signum consisting of broken linear sclerotization surrounded by oval sclerotized ring with blunt dentitions.

Larva. Not preserved.

#### Biology.

Host-plant: *Syzygium acuminatissimum* (Blume) A. DC. (Myrtaceae) (formerly *Acmena acuminatissima*), very closely related to the Australian *Syzygium smithii* (Biffin et al. 2006), a widespread species in the mountains of south Asia, from India and China to New Guinea and the Pacific islands (Chen and Craven 2007). Egg: almost invariably on upperside, almost always on or near midrib. Mine (Fig. 123): a long, very narrow contorted gallery, first half very narrow, running from midrib to leaf margin, or sometimes along midrib, filled with blackish frass, second half much wider, much contorted, often zigzagging, frass compact, black, leaving narrow clear margins; exit-hole on underside, a semicircular hole. Cocoon: ochreous. Occupied mines were collected on 18 and 20 November; they occurred together with abundant mines of a *Heliozela* species.

#### Diagnosis.

Very similar externally to *Pectinivalva (Menurella) acmenae* from Australia, but lacks the pale tornal spot of that species. In the genitalia, the lateral sclerites of the vestibulum are not forked (as they are in *acmenae*), and the apophyses posteriores are distinctly longer than the apophyses anteriores (same length in *acmenae*).

#### Distribution.

Borneo, East Kalimantan: Gunung Lumut.

#### Derivation.

The species name (a noun in apposition) derives from the Greek *xenos* (stranger, foreigner) and *adelpha* (sister) and refers to the close relationship to *acmenae* as well as the great geographical distance between this and other known *Pectinivalva* species.

#### DNA barcode.

RMNH.INS.23738 (holotype), Genbank KC292487 and RMNH.INS.11968 (larva), genbank KC292486, both identical.

#### Remarks.

We choose to describe this species here, even on the basis of a single female, to be able to record the genus from outside Australia. The detailed knowledge of its life history and three DNA markers (including CO1 barcode) will make future association with males straightforward.

### Pectinivalva (Menurella) quintiniae

Hoare & Van Nieukerken sp. n.

urn:lsid:zoobank.org:act:0D0B16C5-745C-433A-9571-169793F72486

http://species-id.net/wiki/Pectinivalva_quintiniae

#### Material examined.

Holotype. ♂, Tullawalal, Lamington National Park, Qld, [UTM: 56J NP188794], la. 19.viii.2004, 900–940 m, [rainforest], emg. 25.ix.-6.x.2004, *Quintinia verdonii*, E.J. van Nieukerken, R.J.B. Hoare, RMNH/EvN no. 2004100, genitalia slide 18720 (anic) (= EJvN 3960). Paratypes. 2♂, 6♀, same data as holotype, genitalia slides ♂: EvN 3736, ♀: EvN 3961, 3993 (anic, rmnh); 2♀, 28.28S,153.07E, Bar Mountain, Border Ranges Nat. Pk, N.S.W., emg. 21–23.viii.2000, *Quintinia verdonii*, C. van den Berg, R.J.B. Hoare (anic, rmnh).

**Additional material:** leafmines from same localities.

#### Description. 

Male (Fig. 15). Wingspan 4.7–4.8 mm. Head: frontal tuft ferruginous; collar inconspicuous, consisting of shining pale grey scales; eyecaps basally white, exteriorly shining grey with violet reflections; antennae shining dark grey, whitish beneath, ca. 35–38 segments. Thorax, tegulae and forewing uniform shining fuscous with strong blue to violet reflections; cilia greyish fuscous. Hindwing unmodified, grey; cilia grey. Underside: forewing dark greyish fuscous; hindwing grey. Abdomen shining dark greyish fuscous; anal tuft inconspicuous, fuscous.

Female (Figs 16, 128). Wingspan 5.0–5.8 mm. Similar to male, but antenna with ca. 27 segments, and forewing rather broader.

Male genitalia (Figs 55–57, 67–69). Capsule ca. 425–465 μm long, ovoid. Anterior edge of vinculum rounded, without excavation. Tegumen rounded, without ventral extensions. Uncus rectangular, bilobed, lobes strongly produced, with ca. 6 setae on each. Gnathos central element long, reaching just beyond uncus, tapering apically. Valva (Fig. 56) ca. 305–320 μm long, rounded caudally; apically fringed with numerous spine-like setae on dorsal surface; pectinifer absent, but apex of valva thickened and well-sclerotized. Long sublateral processes present. Juxta a subrectangular plate. Aedeagus (Figs 57, 69) ca. 455–480 μm long, a curved spine arising towards apex on left. Vesica basally with many broad, short cornuti, grading into field of much larger longer cornuti towards apex.

Female genitalia (Fig. 78, 79, 95–97). Total length ca. 950 μm. T9 produced on each side into prominent anal papillae, each with a group of 5–6 setae. Apophyses anteriores moderately narrow, curved inwards; apophyses posteriores narrow, straight, distinctly shorter than anteriores. Lateral sclerotizations of vestibulum strongly developed, thick, not forked but with outer tooth-like process at ca. ½ length. Ductus spermathecae with 2½ convolutions. Posterior part of corpus broad, folded, without markings; anterior part rounded, with faint pectinations; signum consisting of broken linear sclerotization surrounded by oval sclerotized ring with blunt dentitions; an elongate band of scobination opposite signum.

Larva. Green. Head as in Fig. 108; length of head ca. 410 μm; width ca. 450 μm. Thorax: prothoracic sternite hourglass-shaped; no additional sclerites. Chaetotaxy and spinosity: T2 with 11 pairs of setae (L3 present); otherwise as described for subgenus *Casanovula*. Anal rods not forked posteriorly.

**Figures 104–108.**
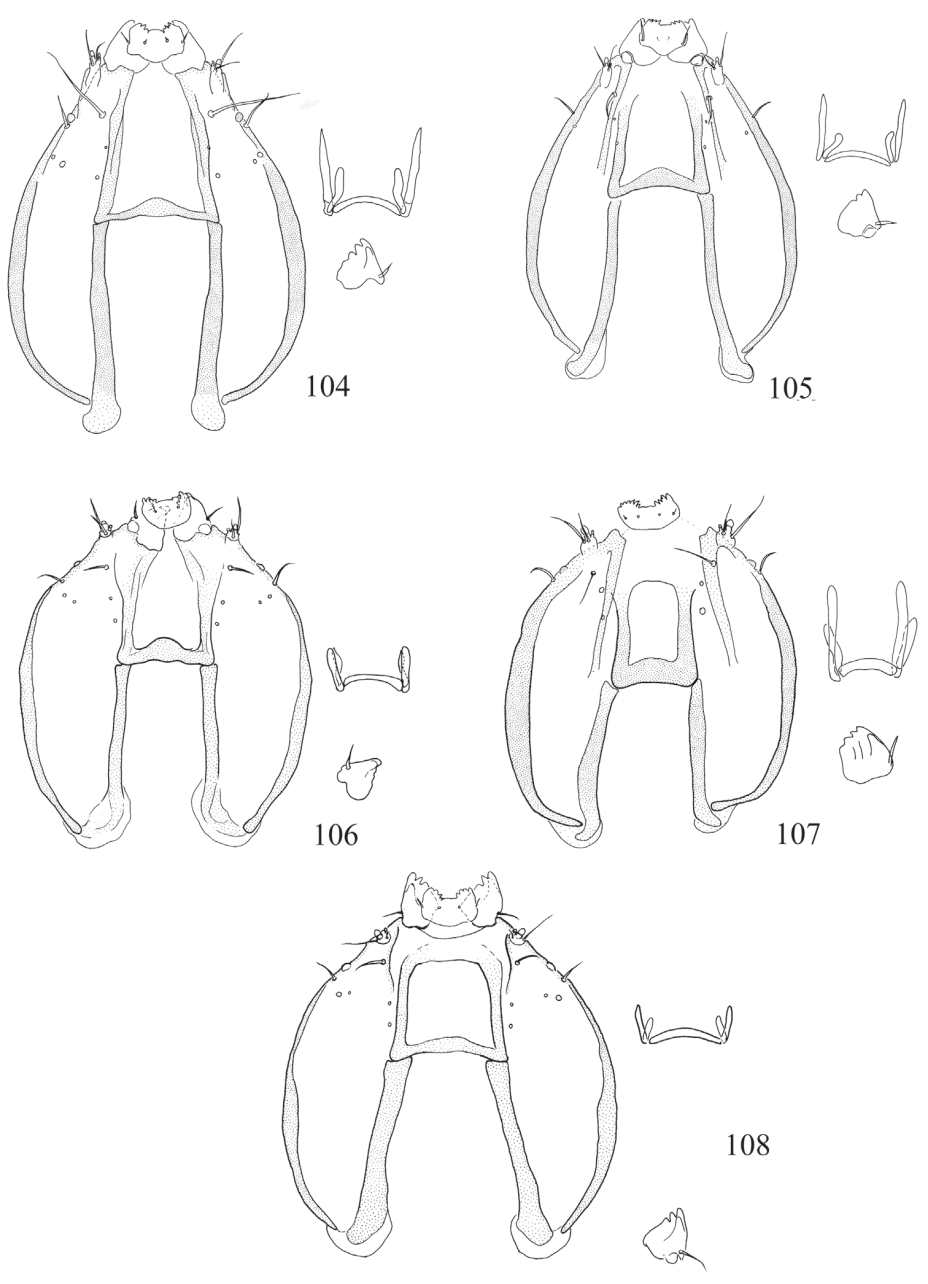
*Pectinivalva* spp., larval heads, dorsal view (head capsule to left, tentorium above right, mandible below right). **104** *Pectinivalva (Pectinivalva)* 138 **105** *Pectinivalva (Casanovula) brevipalpa* **106** *Pectinivalva (Casanovula) minotaurus* **107** *Pectinivalva (Menurella) scotodes* **108** *Pectinivalva (Menurella) quintiniae*.

#### Biology.

Host plant: *Quintinia verdonii* F. Muell. (Paracryphiaceae). Egg: on either side of leaf. Mine (Fig. 122): a long, meandering gallery, central line of blackish frass taking up most of mine width except near end where gallery broadens and frass takes up only ½ width; exit-hole on underside, a semicircular to oval hole. Cocoon: reddish brown. Occupied mines have been collected on 13 July and 19 August.

**Figures 109–114.**
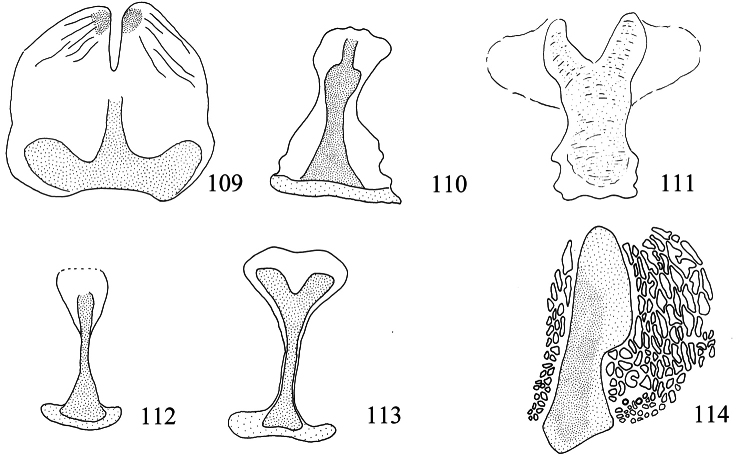
*Pectinivalva* spp., larval prothoracic sclerites. **109–113** Prosternites: **109** *Pectinivalva (Pectinivalva)* 138 **110** *Pectinivalva (Casanovula) brevipalpa* **111** *Pectinivalva (Casanovula) minotaurus* **112** *Pectinivalva (Menurella) scotodes* **113** *Pectinivalva (Enteucha) acmenae*. **114** *Pectinivalva (Pectinivalva)* 5, dorsal sclerite, showing surrounding reticulate cuticle; rings indicate positions of setae D1 and XD1.

**Figures 115, 116.**
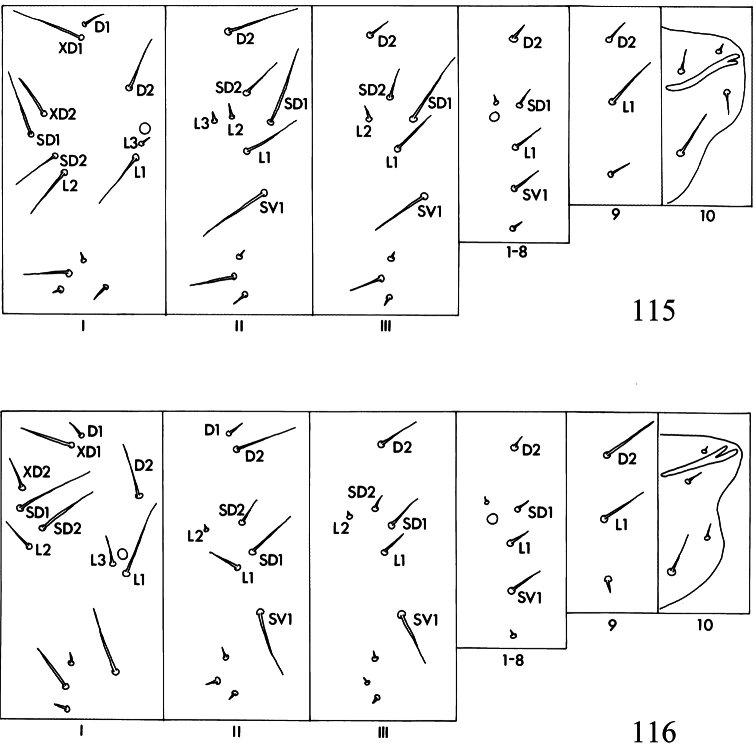
*Pectinivalva* spp., larval chaetotaxy. **115** *Pectinivalva (Pectinivalva)* 138 **116** *Pectinivalva (Casanovula) brevipalpa*.

**Figures 117–124.**
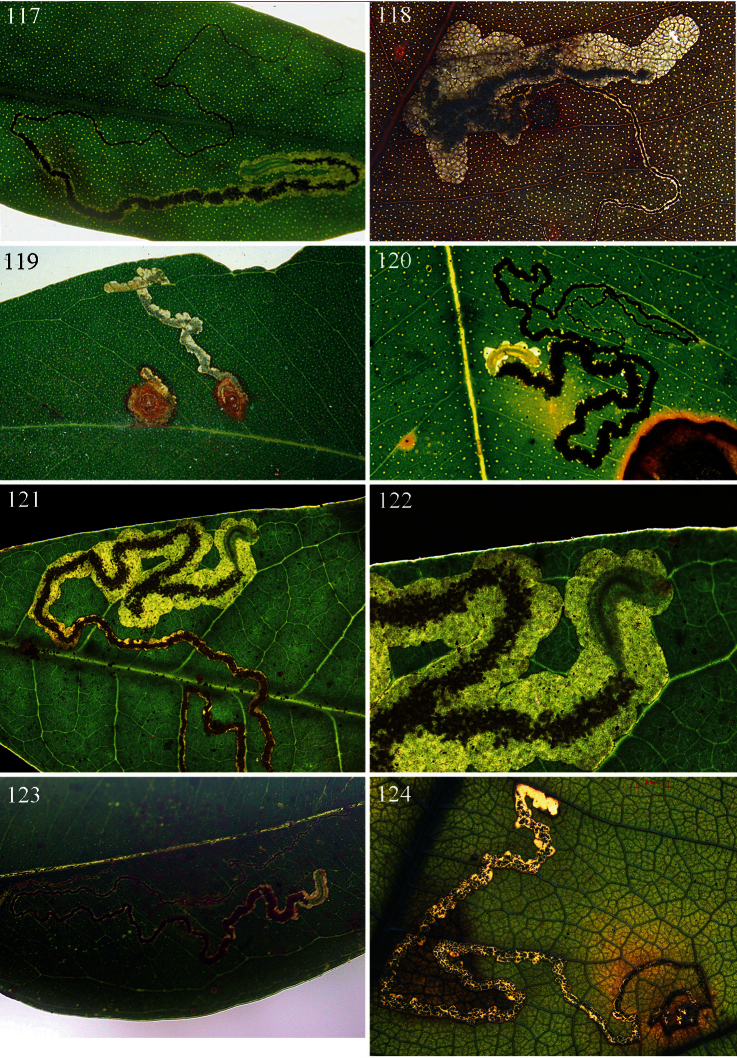
*Pectinivalva* spp., larval leaf-mines. **117** *Pectinivalva (Casanovula) brevipalpa* on *Tristaniopsis collina* **118** *Pectinivalva (Casanovula) minotaurus* vacated mine on *Lophostemon confertus* **119** *Pectinivalva (Menurella) scotodes* on *Eucalyptus pilularis* **120** *Pectinivalva (Menurella) acmenae* on *Syzygium smithii* **121, 122** *Pectinivalva (Menurella) quintiniae* on *Quintinia verdonii* **123** *Pectinivalva (Menurella) xenadelpha* on *Syzygium acuminatissimum* **124** *Pectinivalva (Menurella) tribulatrix* vacated mine on *Rhodomyrtus macrocarpa*.

**Figures 125, 126.**
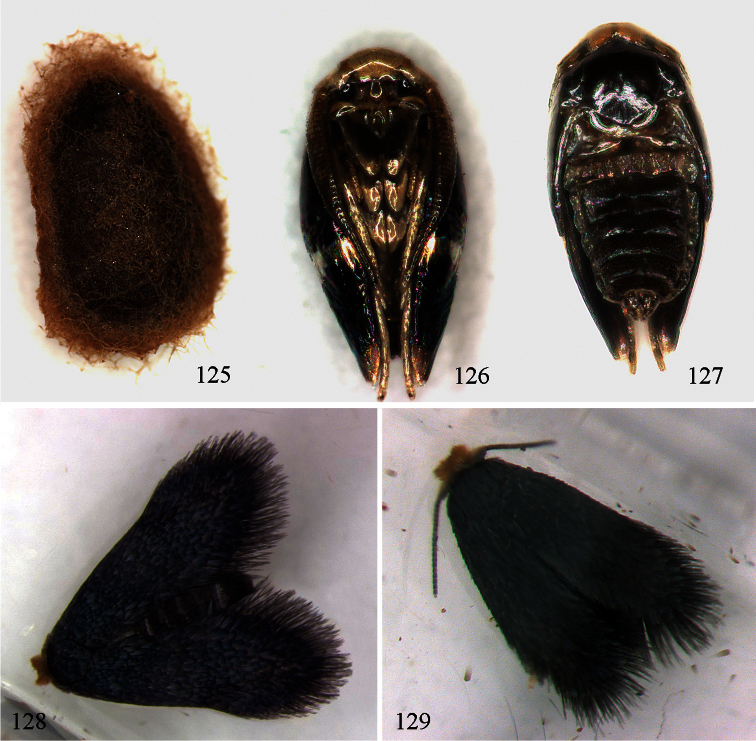
*Pectinivalva (Casanovula)* and *Pectinivalva (Menurella)* spp., cocoon, pupa, live adults. **125** *Pectinivalva (Casanovula) minotaurus* cocoon **126** *Pectinivalva (Casanovula) minotaurus* male pupa (pharate adult), ventral view **127** *Pectinivalva (Casanovula) minotaurus* male pupa (pharate adult), dorsal view **128** *Pectinivalva (Menurella) quintiniae* live adult male **129** *Pectinivalva (Menurella) xenadelpha*, female holotype, live.

#### Diagnosis.

Superficially very similar to *Pectinivalva (Menurella) acmenae*, but lacking the pale tornal forewing spot of that species.

#### Distribution.

Northern N.S.W. (Border Ranges National Park); Southern Queensland, (Lamington National Park).

#### DNA barcode.

RMNH.INS.23736, Genbank KC292482, RMNH.INS.23960 (holotype), Genbank KC292481 and RMNH.INS.23961, Genbank KC292480, with one variable nucleotide.

#### Derivation.

The specific name (a noun in the genitive) is derived from the host-plant genus.

#### Remarks.

Currently this is the only species of *Pectinivalva* known from a host-plant that does not belong to Myrtaceae. *Quintinia* was formerly placed in Escalloniaceae, but is now assigned to the small family Paracryphiaceae (e.g., Winkworth et al. 2008).

### Pectinivalva (Menurella) tribulatrix

Van Nieukerken & Hoare sp. n.

urn:lsid:zoobank.org:act:A3AF88EF-778D-4D82-B333-24DC81DEE3EA

http://species-id.net/wiki/Pectinivalva_tribulatrix

#### Material examined.

Holotype. ♂, Cape Tribulation, Queensland, [UTM: 55K CC365219], la. 23.vii.2004, [coastal rainforest], emg. 8.ix.2004, *Rhodomyrtus macrocarpa*, E.J. van Nieukerken, RMNH/EvN no 2004017, genitalia slide 18721 (anic) (= EvN 3962). Paratype. ♀, same data as holotype, genitalia slide EvN 3963 (rmnh).

**Additional material:** many leafmines from type locality, and 2 km south.

#### Description. 

Male (Fig. 17). Wingspan 3.5 mm, forewing length 1.5 mm. Head: frontal tuft yellow to ferruginous, collar white; eyecaps basally white, exteriorly grey; antennae grey, 25 segments. Thorax and forewing entirely shining grey fuscous, cilia-line indistinct. Hindwing basally wide, grey, with androconial pocket in basal half; cilia grey. Underside: forewing and hindwing dark brown. Abdomen grey brown, with small white anal tufts.

Female (Fig. 18). Wingspan 3.2 mm, forewing length 1.4 mm. Head: as male, but eyecaps shining white, no grey, antennae with 17 segments. Coloration as male, but hindwing narrower, grey. Abdomen shining dark grey, wide blunt abdominal tip.

Male genitalia (Figs 70–72). Capsule ca. 235 μm long, ovoid. Anterior edge of vinculum with shallow excavation. Tegumen rounded, without ventral extensions. Uncus triangular, slightly indented in middle, lobes with ca. 3–4 setae on each. Gnathos central element long, not reaching beyond uncus, parallel edges, rounded tip. Valva ca. 190 μm long, reaching well beyond tegumen, strongly curved; medial edge slightly excavated and ending in obtuse angle; pectinifer consisting of 15–16 broad, blunt elements; dorsal surface towards apex with long setae. Sublateral processes short. Juxta not visible. Aedeagus (Fig. 72) ca. 280 μm long; tubelike sclerite associated with cathrema ca 2/3 aedeagus length, anteriorly bilobed; vesica otherwise with a few small cornuti.

Female genitalia (Fig. 101–103). Total length ca. 335 μm. T9 produced on each side into prominent anal papillae, each with a group of 7 setae. Apophyses anteriores moderately narrow, curved inwards; apophyses posteriores narrow, straight, longer than anteriores. Lateral sclerotizations of vestibulum strongly developed, forked, the bifurcations diverging widely. Ductus spermathecae with 6 convolutions. Corpus small, about as long as wide, folded, covered with many pectinations; signum of concentric bands of fence-like spinules, indistinct.

Larva. Green. Fieldnotes state that it feeds with dorsum upwards, which may be incorrect. Larva not preserved.

#### Biology.

Host-plant: *Rhodomyrtus macrocarpa* Benth., finger cherry (Myrtaceae). Many mines and three larvae were collected on the ca. 20 cm long leaves of seedling shrubs. Egg: on either side of leaf. Mine (Fig. 124): a narrow, long gallery, either completely meandering, or partly straight and following a major vein; frass black, broken and dispersed over total gallery width, not leaving clear margins; edges of gallery not straight, irregular; exit-hole on underside, a semicircular to oval hole. Cocoon reddish brown. Occupied mines have been collected on 22 July.

#### Diagnosis.

One of the smallest *Pectinivalva* species we know, recognised by unmarked greyish fuscous wings, grey edged scape in male and androconial pocket on male hindwing.

#### Distribution.

Northern Queensland, Cape Tribulation.

#### DNA barcode.

RMNH.INS.23962 (holotype), Genbank KC292484 and RMNH.INS.23963, Genbank KC292485, identical.

#### Derivation.

The species name is a noun in apposition, from the Latin *tribulare*, to press: hence *tribulatio*, distress, trouble, *tribulatrix*, one who causes trouble. It refers partly to the type locality (Cape Tribulation), and partly to difficulties the authors encountered in identifying the hostplant.

#### Remarks.

This species stands out from its relatives amongst the ‘derived’ species of *Menurella* (those with broad tooth-like pectinifer elements) in its hostplant *Rhodomyrtus*, which belongs to the tribe Myrteae; other members of this group feed on Eucalypteae.

##### Keys to the subgenera of Nepticulidae known from Australia

The keys presented here are only intended for the identification of nepticulid specimens taken in Australia, and will not necessarily work for material captured elsewhere. They are based on the extensive collection of Nepticulidae in ANIC, with associated larval material. Although the keys should work for all Australian nepticulids so far known, it should be noted that our knowledge of the fauna is still very incomplete and there may possibly be species which will key out incorrectly or not at all.

##### Key to adults, based on external characters and wing venation

**Table:** 

1	Forewing with vein 1+2A unthickened	2
–	Forewing with vein 1+2A thickened	7
2	Forewing with apical suffusion of pale bluish or silver scales	*Roscidotoga*
–	Forewing without apical suffusion of pale bluish or silver scales	3
3	Vein R2+3 of forewing present	*Pectinivalva (Pectinivalva)*
–	Vein R2+3 of forewing absent	4
4	Forewing without bluish or purplish lustre	*Pectinivalva (Menurella)* (part)
–	Forewing with bluish or purplish lustre	5
5	Forewing with transverse fascia	*Pectinivalva (Casanovula)* (part)
–	Forewing without transverse fascia	6
6	Forewing lustre bluish	*Pectinivalva (Menurella)*^1^
–	Forewing lustre purplish	*Pectinivalva (Casanovula)*^2^
7	Collar consisting of lamellate scales	8
–	Collar consisting of piliform scales	9
8	Vein Cu of forewing absent; closed cell present (vestigial)	*Acalyptris*
–	Vein Cu of forewing present; closed cell absent	*Stigmella*
9	Vein M of hindwing 2-branched; male hindwing underside with raised androconia	*Trifurcula (Glaucolepis)*
–	Vein M of hindwing 1-branched; male hindwing underside without raised scales	*Ectoedemia (Fomoria)*

^ 1^ Two species of *Pectinivalva (Menurella)* key out here: *Pectinivalva (Menurella) acmenae* and *Pectinivalva (Menurella) quintiniae*.

^ 2^ One undescribed species of *Pectinivalva (Casanovula)* lacks a fascia and keys out here.

##### Key to adults, based on male genitalia

**Table:** 

1	Gnathos present, not reduced	2
–	Gnathos strongly reduced or absent	*Roscidotoga*
2	Gnathos with 1 central element	3
–	Gnathos with 2 central elements	*Stigmella* (part)
3	Uncus dorsally with 2 well-defined tufts of setae	4
–	Uncus without well-defined tufts of setae	6
4	Uncus pointed, undivided	*Pectinivalva (Pectinivalva)*
–	Uncus bifid	5
5	Cathrema associated with a smooth tubular sclerotization	*Pectinivalva (Menurella)*
–	Cathrema without associated sclerites, or sclerites not forming a smooth tube	*Pectinivalva (Casanovula)*
6	Transverse bar of transtilla membranous or absent	7
–	Transverse bar of transtilla present	8
7	Valvae widely separated at base; aedeagus with carinate processes	*Acalyptris*
–	Valvae close basally; aedeagus lacking carinae	*Trifurcula (Glaucolepis)*
8	Valvae widely separated at base; vinculum with posterior membranous extension; aedeagus with carinae	*Ectoedemia (Fomoria)*
–	Valvae close basally; vinculum unmodified; aedeagus lacking carinae	*Stigmella*^3^

^ 3^ One undescribed *Stigmella* species from South Australia, which has a gnathos with a single central element, keys out here.

##### Key to adults, based on female genitalia

**Table:** 

1	Signum present	2
–	Signum absent	7
2	One signum, not reticulate	3
–	Two reticulate signa	5
3	Signum a pair of concentric ovals of fence-like pectinations	*Pectinivalva (Menurella)* (part)
–	Signum a toothed band	4
4	Lateral sclerites of vestibulum forked or with exterior tooth at ½; signum small, rounded	*Pectinivalva (Menurella)*^4^
–	Lateral sclerites (if present) not forked, no exterior tooth at ½; signum long, narrow	*Pectinivalva (Pectinivalva)*
5	Margins of signa crenulate	Acalyptris
–	Margins of signa smooth	6
6	Vestibulum with complex sclerotizations	*Ectoedemia (Fomoria)*
–	Vestibulum without sclerotizations	*Trifurcula (Glaucolepis)*^5^
7	Apophyses anteriores expanded basally; corpus bursae weakly sclerotized, with diverticulum	*Roscidotoga*
–	Apophyses normal; corpus bursae well or moderately sclerotized; no diverticulum	8
8	Vestibulum with lateral sclerites	*Pectinivalva (Casanovula)*
–	Vestibulum without lateral sclerites	*Stigmella*

^ 4^ Two species of *Pectinivalva (Menurella)* key out here: *Pectinivalva (Menurella) acmenae* and *Pectinivalva (Menurella) quintiniae*.

^ 5^ No females of *Trifurcula* have yet been captured in Australia, and the distinction used here is based on Holarctic members of the genus (see Johansson et al. 1990).

##### Key to the larvae

**Table:** 

1	Antenna 3-segmented	2
–	Antenna 2- or 1-segmented	3
2	Cuticle spinose, with smooth texture; mesothorax with 2 D setae	*Roscidotoga*
–	Cuticle without spines, sculptured; mesothorax with 1 D seta	*Pectinivalva (Pectinivalva)*
3	Antenna 2-segmented	4
–	Antenna 1-segmented	5
4	Hostplant *Lophostemon*, *Tristaniopsis*, or *Melaleuca*	*Pectinivalva (Casanovula)*^6^
–	Hostplant another myrtaceous genus, or *Quintinia*	*Pectinivalva (Menurella)*^6^
5	Sensilla of antenna arranged in a cross	*Stigmella*
–	Sensilla not arranged in a cross	6
6	Mesothorax with 1 D seta	*Ectoedemia (Fomoria)*
–	Mesothorax with 2 D setae	7
7	Labial palpus 2-segmented	*Acalyptris*
–	Labial palpus 3-segmented	*Trifurcula (Glaucolepis)*^7^

^ 6^ No constant morphological differences have been found between larvae of *Pectinivalva (Casanovula)* and those of *Pectinivalva (Menurella)*.

^ 7^ No larva of *Trifurcula* has been found in Australia, and the distinction used here is based on the description of European species in Johansson et al. (1990).

## Supplementary Material

XML Treatment for Pectinivalvinae

XML Treatment for Pectinivalva

XML Treatment for Pectinivalva

XML Treatment for Pectinivalva (Pectinivalva) mystaconota

XML Treatment for Casanovula

XML Treatment for Pectinivalva (Casanovula) brevipalpa

XML Treatment for Pectinivalva (Casanovula) minotaurus

XML Treatment for Menurella

XML Treatment for Pectinivalva (Menurella) scotodes

XML Treatment for Pectinivalva (Menurella) acmenae

XML Treatment for Pectinivalva (Menurella) xenadelpha

XML Treatment for Pectinivalva (Menurella) quintiniae

XML Treatment for Pectinivalva (Menurella) tribulatrix

## Appendix 1. Characters and states for cladistic analysis

All characters were treated as unordered. Figure numbers are given only as exemplars of character states and not all illustrations of each state are necessarily indicated.

1 Shape of male antenna: (0) flagellum of even width throughout; (1) basal flagellar segments expanded and flattened (Figs 24, 25, 29, 30).

2 Sexual colour dimorphism: (0) not pronounced; (1) pronounced (Figs 10, 11).

(3) Forewing pattern of male: (0) more or less unicolorous (Fig. 4); (1) dark with a pale costal streak (Hoare et al. 1997: fig. 1); (2) dark with a pair of opposite pale spots or a pale fascia; (3) metallic with a shining fascia (Fig. 6, 8); (4) metallic with silver spots (Hoare 2000a: figs 1–3).

(4) Androconial scales of male forewing: (0) absent; (1) present as a long dorsal fringe (Hoare et al. 1997: fig. 20).

(5) Forewing venation: (0) R2+3 present (Figs 31, 32); (1) R2+3 absent (Figs 33–36).

(6) Shape of male hindwing: (0) more or less lanceolate (Fig. 33); (1) strongly expanded at base (Figs 31, 35).

(7) Androconial pocket of male hindwing: (0) absent; (1) present (Figs 31, 35).

(8) Male genitalia — anterior extension of vinculum: (0) rounded or smoothly concave (Fig. 39); (1) H-shaped (Fig. 46).

(9) Male genitalia — uncus: (0) undivided (Fig. 39); (1) bifid (Figs 43, 46).

*Notiopostega atrata* has a weakly developed uncus which is very slightly indented apically (Davis 1989: fig. 274): this species has been scored ‘?’ for this character.

(10) Male genitalia — uncus: (0) without well-defined tufts of setae; (1) a pair of well-defined tufts present (Figs 39, 43); (2) tufts present, arising from lobes on dorsal surface of uncus (Hoare et al. 1997: fig. 39).

(11) Male genitalia — central element of gnathos: (0) narrow (at least basally) or triangular (Fig. 39, 43, 46); (1) broad and cordate (Hoare et al. 1997: fig. 38).

The gnathos in *Notiopostega atrata* is reduced and takes the form of a narrow band. *Roscidotoga callicomae* lacks a gnathos. Both species are scored ‘?’ for this character.

(12) Male genitalia — number of elements on pectinifer of valva: (0) 20 or more (Fig. 44, 47); (1) fewer than 20 (Fig. 50); (2) pectinifer strongly reduced or absent (Figs 40, 56).

(13) Male genitalia — shape of pectinifer elements: (0) peg- or spine-like (Figs 44, 53); (1) broad and tooth-like (Fig. 50).

(14) Male genitalia — sublateral processes of transtilla: (0) well-developed (Figs 40, 44); (1) strongly reduced (Hoare et al. 1997: fig. 38).

(15) Male genitalia — cathrema of aedeagus: (0) weakly developed, supported by 2 or 3 interconnected sclerites (Fig. 42) (1) moderately developed, supported by a smooth sclerotized tube (Fig. 51, 72); (2) moderately developed, with 1 short sclerite, or without associated sclerotizations (Fig. 48); (3) absent.

The structure of the cathrema and its associated sclerites in *Pectinivalva* is often obscured by cornuti and hard to observe in conventional slide preparations, and the character coding adopted here is somewhat provisional in nature. Further detailed studies, perhaps of everted vesicas, are desirable.

(16) Female genitalia — lateral sclerites of vestibulum: (0) absent (Fig. 73); (1) present, narrow (Fig. 74); (2) present, strongly developed (Fig. 78).

In *Pectinivalva acmenae*, the lateral sclerites are relatively narrow but have very strongly forked tips (Fig. 77): this species has been tentatively scored (2) for this character.

(17) Female genitalia — pectinations on corpus bursae: (0) extensive (Fig. 74); (1) restricted to a field on the posterior part of the corpus (Fig. 76); (2) very reduced or absent (Fig. 78).

(18) Female genitalia — form of signum: (0) well-sclerotized, sparsely toothed (Fig. 77); (1) well-sclerotized, continuously toothed (Hoare et al. 1997: fig. 73); (2) sclerotization reduced to parallel spinules (Fig. 76); (3) absent (Fig. 75).

(19) Female genitalia — shape of signum: (0) narrow, band-like (Fig. 73); (1) broad, oval (Fig. 76).

(20) Anterior edge of tergite 2 of abdomen: (0) continuously well sclerotized; (1) weakly sclerotized medially (Hoare 2000a: fig. 9).

(21) Larval antenna: (0) 3-segmented (Hoare 2000a: fig. 36); (1) 2-segmented; (2) 1-segmented.

(22) Shape of larval head: (0) anteriorly unmodified, more or less cordate (Fig. 106–108); (1) anteriorly elongate, more or less pyriform (Figs 104, 105).

(23) Posterior lobes of larval head: (0) continuously sclerotized (Hoare 2000a: fig. 29); (1) with sclerotization interrupted (Figs 104–108).

(24) Shape of prothoracic sternite of larva: (0) approximately as long as broad (Fig. 109); (1) much longer than broad (Figs 112, 113).

The larva of *Notiopostega atrata* lacks a prothoracic sternite, and this species has therefore been scored ‘?’ for this character.

(25) Number of mesothoracic D setae of larva: (0) 2 pairs (Fig. 116); (1) 1 pair (Fig. 115).

(26) Spinosity of larval cuticle: (0) cuticle spiny; (1) cuticle lacking spines.

(27) Texture of larval cuticle: (0) smooth; (1) sculptured (Fig. 114).

(28) Exit-hole of larval mine: (0) a small slit or hole (Fig. 118), larva leaves mine to pupate; (1) a large slit, larva pupating in mine.

## Data matrix

0000 00000 11111 1111 12 22222 222

1234 56789 01234 5678 90 12345 678

*Notiopostega* 0000 1000? 0?001 3023 ?0 100?0 000

*Enteucha acetosae* 0030 10000 002?0 3023 ?0 20000 000

*Roscidotoga callicomae* 0040 10000 0?2?0 2023 ?0 00000 000

*Pectinivalva acmenae* 0000 10001 10100 1210 11 10110 000

*Pectinivalva brevipalpa* 1030 10001 10000 0103 ?1 11110 000

*Pectinivalva caenodora* 0010 00000 21001 0101 01 ????? ???

*Pectinivalva commoni* 0000 00000 10000 0100 01 01101 110

*Pectinivalva minotaurus* 1030 10011 10000 2103 ?1 10110 000

*Pectinivalva mystaconota* 0000 01100 102?0 0000 01 ????? ???

*Pectinivalva scotodes* 0101 11101 10110 1212 11 10110 000

*Pectinivalva quintiniae* 0000 10001 102?0 1220 11 20110 000

*Pectinivalva* 2 0000 11101 10100 1212 11 10110 000

*Pectinivalva* 5 0000 00000 10000 0100 01 01101 110

*Pectinivalva* 34 0010 00000 21001 0101 01 01101 110

*Pectinivalva* 91 0020 10101 10110 1212 11 10111 000

*Pectinivalva* 119 0101 11101 10110 1212 11 10010 000

*Pectinivalva* 138 0000 01100 10000 0020 01 01101 110

*Pectinivalva* 163 0000 01100 10000 0020 01 01101 110

*Pectinivalva* 219 0030 10011 10000 2103 ?1 ????? ??1

*Pectinivalva* 226 0030 10011 10000 2103 ?1 10111 001

## Appendix 2. Catalogue of hostplants of *Pectinivalva*

**Myrtaceae**

Lophostemoneae

*Lophostemon ?confertus* (R.Br.) Peter G.Wilson & J.T.Waterh.

*Pectinivalva (Casanovula)* 113

*Lophostemon confertus* (R.Br.) Peter G.Wilson & J.T.Waterh.

*Pectinivalva (Casanovula) minotaurus*, *Pectinivalva (Casanovula)* 41

*Lophostemon suaveolens* (Sol. ex Gaertn.) Peter G.Wilson & J.T.Waterh.

Pectinivalva (Casanovula) minotaurus

Melaleuceae

*Melaleuca citrina* (Curtis) Dum.Cours (= *Callistemon citrinus*)

*Pectinivalva (Casanovula)* 219

*Melaleuca salicina* Craven (= *Callistemon salignum*)

*Pectinivalva (Casanovula)* A98

*Melaleuca squarrosa* Donn ex Sm.

*Pectinivalva (Casanovula)* 226

*Melaleuca viminalis* (Sol. ex Gaertn.) Byrnes (= *Callistemon viminalis*)

*Pectinivalva (Casanovula)* sp EvN2004116

*Melaleuca* sp. (= *Callistemon* sp.)

*Pectinivalva (Casanovula)* 174

Syncarpieae

*Syncarpia glomulifera* (Sm.) Nied.

*Pectinivalva* sp. (RH198) (vacated mines only)

Eucalypteae

*Angophora costata* (Gaertn.) Britten

*Pectinivalva (Menurella)* Q1, *Pectinivalva (Menurella)* 119

*Angophora floribunda* (Sm.) Sweet

*Pectinivalva (Menurella)* Q2, *Pectinivalva (Menurella)* Angophora 1 (CvdB_Loc IV.21.sp1), *Pectinivalva (Menurella)* Angophora 2 (CvdB_Loc IV.21.sp2)

*Corymbia ficifolia* (F.Muell.) K.D.Hill & L.A.S.Johnson

*Pectinivalva (Menurella)* 127

*Corymbia maculata* (Hook.) K.D. Hill & L.A.S. Johnson

*Pectinivalva (Menurella)* 29

*Corymbia torelliana* (F.Muell.) K.D.Hill & L.A.S.Johnson (=*Eucalyptus torelliana*)

*Pectinivalva* sp. (RH114) (vacated mines only)

*Eucalyptus acmenoides* Schauer

*Pectinivalva* sp. (RH158) (vacated mines only)

*Eucalyptus blakelyi* Maiden

Pectinivalva (Menurella) scotodes

*Eucalyptus ?blakelyi* Maiden

*Pectinivalva (Pectinivalva)* 78

*Eucalyptus bridgesiana* R.T.Baker

*Pectinivalva (Menurella)* 91, *Pectinivalva (Menurella)* 92, *Pectinivalva (Menurella)* 123, *Pectinivalva (Pectinivalva)* 138, *Pectinivalva (Pectinivalva)* 148a, *Pectinivalva (Pectinivalva)* 163

*Eucalyptus carnea* R.T. Baker

*Pectinivalva (Menurella) scotodes*, *Pectinivalva (Pectinivalva)* 225

*Eucalyptus cyanophylla* Brooker

*Pectinivalva (Menurella)* 76

*Eucalyptus cypellocarpa* L.A.S. Johnson

*Pectinivalva (Pectinivalva)* 89, *Pectinivalva (Pectinivalva)* 90

*Eucalyptus* ?*delegatensis* R.T. Baker

Pectinivalva (Pectinivalva) commoni

*Eucalyptus dives* Schauer

*Pectinivalva (Menurella)* 91

*Eucalyptus elata* Dehnh.

*Pectinivalva (Pectinivalva)* 100

*Eucalyptus ?globoidea* Blakely

*Pectinivalva (Pectinivalva)* 153

*Eucalyptus globulus* Labill.

*Pectinivalva (Menurella)* Q3, *Pectinivalva (Menurella)* 91, *Pectinivalva (Pectinivalva)* A87, *Pectinivalva (Pectinivalva)* 148a

*Eucalyptus goniocalyx* F.Muell. ex Miq.

*Pectinivalva (Pectinivalva)* 148b

*Eucalyptus grandis* W. Hill

*Pectinivalva (Pectinivalva)* 148a

*Eucalyptus leptophylla* F.Muell. ex Miq.

*Pectinivalva (Menurella)* 91

*Eucalyptus macarthurii* H.Deane & Maiden

*Pectinivalva (Pectinivalva)* A87, *Pectinivalva (Menurella)* 150

*Eucalyptus mannifera* Mudie

*Pectinivalva (Pectinivalva)* 78, *Pectinivalva (Menurella)* 151

*Eucalyptus melliodora* A.Cunn. ex Schauer

*Pectinivalva (Menurella)* 23, *Pectinivalva (Menurella)* 136

*Eucalyptus microcorys* F. Muell.

*Pectinivalva (Menurella)* 23

*Eucalyptus ?muelleriana* A.W. Howitt

*Pectinivalva (Menurella)* 91

*Eucalyptus nitida* Hook. f.

*Pectinivalva (Menurella)* 227

*Eucalyptus oleosa* F.Muell. ex Miq.

*Pectinivalva* sp. (RH169) (vacated mines only)

*Eucalyptus pilularis* Sm.

*Pectinivalva (Menurella) scotodes*, *Pectinivalva (Menurella)* 91

*Eucalyptus polyanthemos* Schauer

*Pectinivalva (Menurella)* 21

*Eucalyptus rubida* H.Deane & Maiden

*Pectinivalva (Pectinivalva)* 139

*Eucalyptus saligna* Sm.

*Pectinivalva (Menurella)* KM7

*Eucalyptus ?saligna* Sm.

*Pectinivalva (Pectinivalva)* 5

*Eucalyptus socialis* F.Muell. ex Miq.

*Pectinivalva* sp. (RH168) (vacated mines only)

*Eucalyptus tereticornis* Sm.

*Pectinivalva (Pectinivalva)* 68

*Eucalyptus viminalis* Labill.

*Pectinivalva (Menurella)* 143, *Pectinivalva (Pectinivalva)* 78, *Pectinivalva (Pectinivalva)* 142

Leptospermeae

*Leptospermum ?glaucescens* S.Schauer

*Pectinivalva (Menurella)* 211

*Leptospermum laevigatum* (Gaertn.) F.Muell.

*Pectinivalva (Menurella)* A85

Leptospermum morrisonii

*Pectinivalva (Menurella)* 189

*Leptospermum scoparium* Joy Thomps.

*Pectinivalva (Menurella)* 216

*Leptospermum trinervium* (Sm.) Joy Thomps.

*Pectinivalva (Menurella)* 2, *Pectinivalva (Menurella)* 175

Kanieae

*Tristaniopsis collina* Peter G. Wilson & Waterhouse

Pectinivalva (Casanovula) brevipalpa

Syzygieae

*Syzygium acuminatissimum* (Blume) A. DC. (= *Acmena acuminatissima*)

Pectinivalva (Menurella) xenadelpha

*Syzygium ingens* (F.Muell. ex C.Moore) Craven & Biffin (= *Acmena brachyandra*)

*Pectinivalva (Menurella)* cf *acmenae* (EvN2004099) (vacated mines only)

*Syzygium smithii* (Poir.) Nied. (= *Acmena smithii*)

Pectinivalva (Menurella) acmenae

Myrteae

*Gossia bidwillii* (Benth.) N.Snow & Guymer (= *Austromyrtus bidwillii*)

*Pectinivalva* sp. (RH46) (vacated mines only)

*Pilidiostigma tropicum* L.S.Sm.

*Pectinivalva* sp. (RH106) (vacated mines only)

*Rhodomyrtus macrocarpa* Benth.

Pectinivalva (Menurella) tribulatrix

**Paracryphiaceae**

*Quintinia verdonii* F. Muell.

Pectinivalva (Menurella) quintiniae

## Appendix 3

Specimen locality data of *Pectinivalva* Scoble (Lepidoptera: Nepticulidae) and outgroup taxa: examined specimens, DNA vouchers and host records. (doi: 10.3897/zookeys.278.4743.app) File format: Microsoft Excel (xlsx). **Explanation note:** The records comprise all material studied, including rearing records of species that have not been treated and voucher specimens for DNA barcoding. We have treated specimens with a genitalia slide or other preparation and DNA vouchers as individual records for each species. Other records combine all adults from a single rearing or other single collection event. While compiling this datasheet, the authors no longer had direct access to the ANIC collection in Canberra, and in some cases our notes were not sufficient to enter all fields. This explains why for some slides ‘sex unknown’ is given, and uncertainties about exactly how many specimens were collected on a given date.
